# On the distribution and interpretation of voice in Greek anticausatives

**DOI:** 10.3389/fpsyg.2023.1068058

**Published:** 2023-02-23

**Authors:** Evripidis Tsiakmakis, Joan Borràs-Comes, M. Teresa Espinal

**Affiliations:** ^1^Department of Filologia Catalana/Center for Theoretical Linguistics, Autonomous University of Barcelona, Barcelona, Spain; ^2^University of Barcelona, Barcelona, Catalonia, Spain

**Keywords:** expletive voice, anticausatives, semantic redundancy, Greek, experimental results

## Abstract

This paper provides experimental evidence in support of the view that Greek does not have three productive morphological classes of anticausative verbs, but only two: the class of verbs that bear non-active voice morphology and the class of verbs that are morphologically active. Across two experiments, native Greek speakers are found to prefer for each anticausative verb either non-active or active voice morphological marking, in the presence or absence of explicit contextual information. It is also shown experimentally that native speakers prefer an interpretation that involves a specific cause for all anticausatives, especially when the existence of such a cause is favored by the contextual setting. Our empirical findings are consistent with the view that the Voice Phrase that is realized as non-active voice morphology in Greek anticausatives is expletive. From a theoretical perspective, we analyze the expletiveness of this Voice projection as the result of semantic redundancy: the Voice head of Greek anticausatives combines with a *v* head that encodes a redundant cause meaning component and is, therefore, interpreted merely as introducing an identity function.

## 1. Introduction

Across languages, there are groups of verbs that exhibit what is known as the causative alternation (Levin, 1993; Levin and Rappaport Hovav, 1995; Schäfer, 2008; Rappaport Hovav, 2014, a.o.): they display an intransitive variant describing a change of state and a transitive variant describing a situation where somebody causes this change of state. The former, exemplified by (1a), is dubbed the anticausative variant, while the latter, exemplified by (1b), is labeled as the causative variant.

**Table:** 

(1)	a.	The door *opened*.	*Anticausative*
	b.	Jane *opened* the door.	*Causative*

In the present paper we will be focusing on the anticausative part of the alternation and on one language only, namely Greek.

Greek verbs are morphologically marked for tense, aspect, voice, and agreement with the subject (Triantafyllidis, 1941; Holton et al., 1997, among many others; see also Ralli, 2005). Especially regarding voice, a dual distinction is made between *active voice* morphology (2a) and *non-active voice* morphology (2b).^1^

**Table:** 

(2)	a.	ziyi*-se*	*Active voice morphology*
		weigh-perf.act.past.3.sg	
		‘he weighed’	
	b.	ziyi*-stike*	*Non-active voice morphology*
		weigh-perf.nact.past.3.sg	
		‘he was weighed’	

In the framework of Alexiadou et al. (2015), Greek voice morphology, as exemplified in (2), is mapped onto voice syntax in the following way: Non-active voice marking (2b) is uniformly considered as the realization of a syntactic non-active Voice Phrase (VoiceP), that does not project a specifier (Embick, 1997) and appears in intransitive construals like (3) below.

**Table:** 

(3)	To	pedhi	vafti*stike*	xtes.
	the	kid	baptize.perf.nact.past.3.sg	yesterday
	‘The kid was baptized yesterday.’			

On the other hand, active voice marking (2a) may correspond to two different structures: (i) the projection of an active VoiceP that does project a specifier and appears in transitive construals (4), or (ii) the absence of a VoiceP altogether, in the case of unaccusatives for instance (5).^2^

**Table:** 

(4)	O	Kostas	eli*se*	ton	ghrifo.
	the	Kostas	solve.perf.act.past.3.sg	the	riddle.
	‘Kostas solved the riddle.’				
(5)	To	luludhi	anthi*se*.		
	the	flower	blossom.perf.act.past.3.sg		
	‘The flower blossomed.’				

Regarding specifically anticausative verbs, Alexiadou et al. (2015); see also Theophanopoulou-Kontou, 2000; Tsimpli, 2006) claim that in Greek they can be divided into three morphologically distinct classes: verbs of Class A bear non-active voice morphological marking (6), verbs of class B are morphologically active (7), and verbs of Class C can appear with either active or non-active voice morphology (8).^3^

**Table:** 

(6)	To	karavi	vithi*stike*.		*Class A*
	the	boat	sank.nact^4^		
	‘The boat sank.’				
(7)	I	porta	anik*se*.		*Class B*
	the	door	opened.act.		
	‘The door opened.’				
(8)	To	kotetsi	gremi*stike*/	gremi*se.*	*Class C*
	the	hencoop	crumbled.nact	crumbled.act.	
	‘The hencoop crumbled.’				

Under the morphology-syntax mapping adopted by Alexiadou et al. (2015), the three morphological classes of anticausative verbs above can be associated with corresponding syntactic classes: Class A verbs are characterized by the projection of (non-active) VoiceP, Class B verbs are characterized by the absence of (active) Voice, and Class C verbs are identified *via* the optionality of the projection of a (non-active) VoiceP.

The question is readily raised whether the three morphological and/or syntactic classes of anticausative verbs are also mapped onto different meanings. Concretely put, do anticausatives of one class receive a systematically different interpretation from anticausatives of the other classes? And in the same vein, what is the interpretation of voice in Greek anticausatives? There are two ways to address these questions: either by comparing the meaning of Class A and Class B verbs (projection of a VoiceP in a verb X vs. non-projection of VoiceP in a verb Y), or by comparing Class C anticausative minimal pairs [projection of a VoiceP in a verb X vs. non-projection of VoiceP in the same verb X; see example (8)].

Researchers interested in the topic have mostly followed the latter alternative. Alexiadou and Anagnostopoulou (1999, 2004), for example, report that Class C anticausatives with active voice morphology are incompatible with adverbs such as *endelos* ‘completely’, while their non-actively marked counterparts are not:

**Table:** 

(9)	a.	To	kotetsi	gremi*se*	(^#^*endelos*).
		the	hencoop	crumbled.act	completely
	b.	To	kotetsi	gremi*stike*	*endelos*
		the	hencoop	crumbled.nact	completely
		‘The hencoop crumbled completely.’			

The authors take the contrast to suggest that morphologically non-active Class C verbs [such as *gremistike* in (9b)] can convey either partial or complete change of state, while the ones bearing active voice morphology receive only partial-change interpretation [hence the ill-formedness of (9a)].

It is important to note that Alexiadou and Anagnostopoulou (1999, 2004) report their own judgments. But even if we accept that their generalization holds, it is hard to link a partial vs. complete change of state interpretative distinction to the presence (non-active voice morphology) vs. the absence of a VoiceP (active voice morphology), the semantics of which have been related mostly to the introduction (or existential binding) of the external argument since Kratzer (1996).

Oikonomou (2014) focuses on a different contrast attributed to Class C anticausative verbs, as exemplified in (10):

**Table:** 

(10)	a.	To	ftero	tu	aftokinitu	mu	^#^tsalako*se*/	tsalako*thik*e.^5^
		the	fender	of.the	car	mine	crumpled. act	crumpled. nact.
		‘The fender of my car crumpled.’						
	b.	I	fusta	mu	tsalako*se*/	tsalako*thik*e.		
		the	skirt	mine	crumpled. act	crumpled. nact		
		‘My skirt crumpled.’						
						[Oikonomou, 2014: 45, exs. (84a, b)]		

According to the author’s intuitions, Class C anticausatives with active voice morphology are infelicitous when the described change of state is violently caused by an external initiator –as in the crumpling of a car fender (10a). In the case of not externally (nor violently) caused changes, as is the crumpling of a skirt (10b), both active and non-active Class C verbs are appropriate.

Ιt appears that Oikonomou (2014) offers a feasible answer to what the interpretation of voice in Greek anticausatives may be: it allows the external cause that brings about the described change of state to enter the derivation as a semantic argument.^6^ However, this approach should be taken with caution for two reasons: First, this author also builds on judgments not proven to be shared by other Greek speakers. Second, her proposal is developed around something very close to the internal vs. external causation distinction (Levin and Rappaport Hovav, 1995), the grammatical relevance of which has been cast under doubt (Rappaport Hovav, 2020).

A radically different proposal is put forth by Alexiadou et al. (2015); see also Oikonomou and Alexiadou, 2022), who claim that no interpretative distinction can be systematically mapped to the presence vs. absence of a VoiceP in Greek anticausatives. The authors argue that, instead, actively and non-actively marked Class C anticausatives are semantically equivalent as regards their event structure, and postulate that the VoiceP projected in Greek anticausatives with non-active morphology is expletive (Schäfer, 2008; Wood, 2014, 2015); it is interpreted as introducing an identity function (Winter, 2016) over predicates of events.^7^

Putting together the above, the interpretation of voice in Greek anticausatives has given rise to an unresolved debate. Setting the *partial* vs. *complete chang*e discussion (Alexiadou and Anagnostopoulou, 2004) at the side, as its relevance was refuted by the very authors that opened it (Alexiadou et al., 2015), we are left with the *external causation* hypothesis (Oikonomou, 2014) on the one hand, and the *expletiveness* hypothesis (Schäfer, 2008), on the other. Still, all these previous studies do not support their respective analyses with broad experimental data that report (i) on the acceptability and naturalness judgments that Greek native speakers attribute to Class C anticausative verbs with either active or non-active voice morphology, and (ii) on the interpretation of these verbs. The study presented here pursues two goals: (i) to fill the gap in the empirical argumentation by offering novel experimental evidence on the distribution and meaning of voice in Greek anticausatives, and (ii) to explore an analysis that explains the compatibility of an expletiveness approach to Voice with an (external) causation approach to anticausatives.

The paper is structured as follows: Section 2 consists in a detailed description of our experimental study. In Section 3 we discuss the empirical and theoretical consequences of our findings regarding the distribution and interpretation of anticausative voice in Greek. Section 4 concludes the paper.

## 2. The experimental study

An experimental study was designed in an attempt to gather evidence concerning the speaker’s choice of non-active vs. active voice morphological marking in Greek anticausatives, and contribute to settling the debate laid out in the Introduction. Precisely, we aimed to examine the hypothesis put forth in Alexiadou et al. (2015) that postulates that the Voice Phrase of Greek non-actively marked anticausatives is semantically expletive.

In order to test this hypothesis, we carried out our experimental study in two steps. First, we conducted a very simple acceptability judgment task with intransitive sentences whose main verb was anticausative. Second, we investigated whether the speaker’s choice and interpretation of actively vs. non-actively marked anticausative verbs was sensitive to contextual information. Concretely, the presence of brief contextual settings would allow us to test whether these settings might condition the speakers’ preferences for (i) some specific morphological setup and (ii) a cause vs. no-cause interpretation of the anticausative event.

In what follows, we present the two experiments that formed the basis of our study.

### 2.1. Experiment 1

As anticipated, the linguists interested in the interpretative contribution of voice in Greek anticausatives focused mostly on Class C verbs, that is the set of verbs that allegedly form both actively and non-actively marked anticausatives. We therefore thought that our focus should be also on the nature of Class C verbs, in order to find out whether this class has the same status in the grammar of native speakers as Classes A (only non-actively marked anticausatives) and B (only actively marked anticausatives). Our research question in the very first step of our study was whether native speakers have a clear preference for associating a subset of Class C verbs with active morphology and a different subset with non-active morphology or they do not. Two patterns seemed likely: Participants might not differentiate between the two morphological options; this would suggest that Class C, including verbs that randomly display active or non-active voice morphology, is productive in the grammar of Greek. Alternatively, participants might systematically choose active voice morphology for some verbs and non-active voice morphology for a different group of verbs; this would indicate that Class C is not productive.

To the above end, actively marked Class C anticausatives were contrasted directly to their counterparts with non-active voice marking. Participants had to rate the acceptability of sentences that featured either one form or the other.^8^ The survey was administered *via* Alchemer.

#### 2.1.1. Participants

Experiment 1 was voluntarily completed by 90 native speakers of Greek (44 male, 44 female, 2 other; mean age 28.91 years, SD = 3.99), recruited *via* Facebook and other social media platforms.

#### 2.1.2. Materials

For the materials of this experiment, 10 anticausative verbs that allegedly belong to Class C (Alexiadou et al., 2015) were used: *rayizo* ‘crack’, *madhao* ‘pluck’, *zarono* ‘wrinkle’, *erimono* ‘desert’, *zesteno* ‘heat’, *lerono* ‘sully’, *dhialio* ‘disperse’, *gremizo* ‘crumble’, *tsalakono* ‘crumple’, *skizo* ‘tear’. Each verb appeared in its active and its non-active voice morphological guise in affirmative sentences and as part of the same sentential environment. This led to a total of 20 critical experimental items. We provide two of the minimal item pairs featured in Experiment 1 below, along with the English translation. See Appendix for the full list of critical items.

**Table:** 

(11)	a.	To	plithos	dhieli*se*.
		the	crowd	dispersed.act.
	b.	To	plithos	dhiali*thike.*
		the	crowd	dispersed.nact.
		‘The crowd dispersed.’		
(12)	a.	I	tixi	rayi*san*.
		the	walls	cracked.act.
	b.	I	tixi	rayi*stikan.*
		the	walls	cracked.nact.
		‘The walls cracked.’		

Recall that, if Class C is productive in the grammar of Greek native speakers, participants are not expected to distinguish between the active (11a, 12a) and the non-active voice morphology (11b, 12b). By contrast, if speakers have a clear preference for one of the two forms of each verb and assign significantly different acceptability ratings to the members of each pair in our experiment, this can be regarded as evidence in support of the view that Class C is not productive.

The set of materials for Experiment 1 was complemented with 20 control items: 10 sentences featured Class A anticausative verbs, i.e., anticausatives that obligatorily bore non-active voice morphology (*metavalome* ‘change’, *vithizome* ‘sink’, *anatrepome* ‘turn over’, *peristrefome* ‘rotate’, *mionome* ‘diminish’, *epidhinonome* ‘deteriorate’, *veltionome* ‘improve’, *anaptisome* ‘grow’, *trelenome* ‘go crazy’, *ekrighnime* ‘explode’); additionally, 10 sentences featured Class B anticausative verbs, i.e., anticausatives that were necessarily marked as active (*alazo* ‘change’, *vuliazo* ‘sink’, *anapodhoyirizo* ‘turn over’, *yirizo* ‘rotate’, *lighostevo* ‘diminish’, *xiroterevo* ‘deteriorate’, *kaliterevo* ‘improve’, *meghalono* ‘grow’, *salevo* ‘go crazy’, *skao* ‘explode’). The control items were included in order to confirm the participants’ competence to evaluate voice morphology independently of the specific questions that our experiment addressed. We further made sure that each Class A verb used had a synonym in the group of Class B verbs chosen for the experiment, which allowed us to maintain a control item design similar to the one of the criticals. Two minimal item pairs pertaining to the controls are given below, translated into English (see Appendix for the set of items).

**Table:** 

(13)	a.	I	orasi	tu	veltio*thike*.	*Class A.*
		the	vision	his	improved.nact.	
	b.	I	orasi	tu	kaliterep*se*.	*Class B.*
		the	vision	his	improved.act.	
		‘His vision improved.’				
(14)	a.	To	politiko	skiniko	metavli*thike*.	*Class A.*
		the	political	scenery	changed.nact.	
	b.	To	politiko	skiniko	alak*se*.	*Class B.*
		the	political	scenery	changed.act.	
		‘The political scenery changed.’				

We gave participants the following instructions: “In what follows, you will be presented with a set of sentences. Each sentence is followed by a scale. We ask you to use this scale to rate how good each sentence is in your opinion (left extreme = bad, right extreme = good).”

Our participants rated the total of items, producing 40 ratings each (20 critical items +20 control items). A sum of 3,600 responses (90 participants × 40 ratings) were statistically analyzed.

#### 2.1.3. Procedure

Participants completed Experiment 1 using their personal computer or smart device. First, they had to read the instructions and answer a brief questionnaire concerning their sociolinguistic background (see Appendix). Then the main task started, in which they were asked to read sentences in isolation (i.e., in total absence of context) and use a scale to report whether they found each sentence good or bad.

The items were randomized. An example follows of what participants saw on their screens, translated into English.

**Table:** 

(15)	To	pandeloni	skistike.	
	the	pants	teared.nact	
	‘The pants teared.’			
	kaki	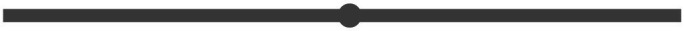		kali
	‘bad’			‘good’

The median duration of the experiment was 6′ 93″.

Before we present the results of this acceptability judgment task, we introduce in Section 2.2 a modified variant of Experiment 1, one that required the evaluation of sentences against specific contextual settings and aimed to investigate not only the speaker’s preference for active vs. non-active voice morphology but also the meaning ascribed to anticausative verbs bearing this morphology.

### 2.2. Experiment 2

Our second experiment aimed at getting additional evidence concerning the original hypothesis we investigated, according to which non-active voice in Greek anticausatives is semantically expletive. Recall that while Experiment 1 meant to investigate whether speakers have a clear preference for one of the two anticausative variants of so-called Class C verbs in the absence of contextual information, the purpose of Experiment 2, which was also based on an acceptability judgment task, was to explore whether the acceptability of active vs. non-active forms and their interpretation differ when participants are forced to directly contrast the actively and the non-actively marked anticausative of a Class C verb against different contextual settings.

Given that there are infinite aspects of the contextual setting that could possibly affect the preference for and interpretation of active or non-active voice marking on anticausative Class C verbs, and taking into account the previous literature (namely Oikonomou, 2014), we identified the saliency of an external cause of the described change of state as the most relevant factor. Concretely, our research question at this second stage of our study was twofold; we meant to test (i) whether the speakers’ preference for active or non-active voice morphology on a Class C anticausative verb is sensitive to the (non-)saliency of an external cause linguistically phrased in the description of the situational environment, and (ii) whether the speakers’ interpretation of an anticausative as involving a specific cause or not is sensitive to the voice morphology of the verb or the description of a (non-)salient external cause linguistically phrased in the contextual setting.

As regards the first part of our research question, there were two possibilities: Speakers might not systematically associate the existence of a salient external cause with one voice morphology or the other; this would suggest that no connection can be established between voice morphology and the presence or absence of cause-related information. (Note that, in that case, we would expect to obtain a morphological preference pattern close to that obtained from Experiment 1). Alternatively, speakers might relate systematically the non-active vs. active voice morphology distinction to the (non-)salient external cause distinction, regardless of the specific verb; this would imply that the choice of one morphological variant over the other reflects information regarding the cause of the described change.

Concerning the second part of our research question, we again considered two alternatives: Participants might interpret or accommodate a specific cause for the anticausative event when the context makes such a cause salient, but deny the existence of a specific cause when the contextual setting is not sufficiently informative or restrictive in this respect. This would suggest that speakers base their interpretation on contextual information. Alternatively, participants might interpret that there is a specific cause when the verb bears non-active voice morphology but prefer an interpretation where no concrete cause for the described change of state is specified for verbs with active voice marking. This would suggest that speakers are more sensitive to morphological than contextual information when interpreting anticausatives.

Given this twofold research question, we tested the active vs. non-active morphology distinction in Class C anticausatives against two types of contexts: (i) a context that introduced *via* linguistic means an external cause or initiator interpreted as causing the described change of state (e.g., a fire, a storm, an earthquake) –let us call this the *overt cause* context, and (ii) a context where no such external initiator bringing about the change of state was explicitly introduced in the situation description –let us dub this the *non-overt cause* context.^9^ In Experiment 2, participants were asked to read some texts consisting of brief contexts followed by short sentences, rate the naturalness of the sentences, and provide judgments on their possible interpretations: a *cause interpretation* that identified a specific external cause in the described event, and an interpretation that negated the existence of a specific cause, labeled as *no-cause interpretation* merely for ease of reference. This survey was also administered *via* Alchemer.

#### 2.2.1. Participants

A total of 76 participants (20 male, 55 female, 1 other; mean age 29.91 years, SD = 5.69), all native speakers of Greek, completed Experiment 2. In this case, too, we used Facebook and other social media platforms to recruit participants.

#### 2.2.2. Materials

For the critical items of Experiment 2 we used the same 10 verbs as in Experiment 1; in fact, we used exactly the same sentences. However, this time we further introduced a contextual setting. Accordingly, each verb appeared in its active and its non-active variant, and each variant was preceded by an overt cause context and a non-overt cause context, bringing our critical items to a total of 40 (see Appendix for the full list of critical items). Below we show how our example (12) from Experiment 1 was adapted for the purposes of Experiment 2. The contexts are here translated into English for the reader’s convenience.

**Table:** 

(16)	a.	Overt cause context – Active voice.		
		[The earthquake was rather strong. The seven-storey building was deeply shaken.]		
		I	tixi	rayi*san*.
		the	walls	cracked.act.
		‘The walls cracked.’		
	b.	Non-overt cause context – Active voice.		
		[After seventy years, the building was now uninhabitable. The signs were obvious.]		
		I	tixi	rayi*san*.
		the	walls	cracked.act.
		‘The walls cracked.’		
	c.	Overt cause context – Non-active voice.		
		[The earthquake was rather strong. The seven-storey building was deeply shaken.]		
		I	tixi	rayis*tik*an.
		the	walls	cracked.nact.
		‘The walls cracked.’		
	d.	Non-overt cause context – Non-active voice.		
		[After seventy years, the building was now uninhabitable. The signs were obvious.]		
		I	tixi	rayis*tik*an.
		the	walls	cracked.nact.
		‘The walls cracked.’		

The context in (16a,c), in contrast with the context in (16b,d), stated explicitly an external initiator for the change of state described in the test sentence; it was the strong earthquake that is meant to have caused the walls to crack. It is in this sense that we considered the former an overt cause context, contrasting with the latter that included no overt initiator; the cracking of the walls is attributable to the effect of time or the tendency of all material things to perish. If anticausative voice is indeed expletive (Alexiadou et al., 2015), then the (non-)overtness of the cause is not expected to interact with the speakers’ preference for active or non-active voice morphology. In other words, no significant differences between (16a) and (16b) on the one hand, and (16c) and (16d) on the other, are to be found. It is further expected that the difference between (16a,b) and (16c,d) will mirror the findings of Experiment 1.

Having in mind a potential comparison of the results from Experiment 1 and Experiment 2, we decided to keep in the latter the set of control items used for the former (Class A and B anticausatives). The controls were also adapted by including contextual information, so as to have a structure parallel to the criticals. Consider the reformulation of example (13) in (17), with the contexts translated into English again here for expository purposes. See Appendix for the set of items used in Experiment 2 in their original form.

**Table:** 

(17)	a.	Overt cause context – Active voice.			
		[Grandpa was complaining that he could not see from one eye. I got him a basic collyrium from the pharmacy.]			
		I	orasi	tu	kaliterep*se*.
		the	vision	his	improved.act.
		‘His vision improved.’			
	b.	Non-overt cause context – Active voice.			
		[The puppy was so young he could not even see. Weeks passed.]			
		I	orasi	tu	kaliterep*se*.
		the	vision	his	improved.act.
		‘His vision improved.’			
	c.	Overt cause context – Non-active voice.			
		[Grandpa was complaining that he could not see from one eye. I got him a basic collyrium from the pharmacy.]			
		I	orasi	tu	veltio*thike*.
		the	vision	his	improved.nact.
		‘His vision improved.’			
	d.	Non-overt cause context – Non-active voice.			
		[The puppy was so young he could not even see. Weeks passed.]			
		I	orasi	tu	veltio*thike*.
		the	vision	his	improved.nact.
		‘His vision improved.’			

Recall that the research question that we addressed *via* Experiment 2 also had a second part, related to whether participants interpret a specific cause when encountered with anticausative event descriptions or not. Aiming at exactly this, every test sentence was followed by two possible interpretations: one that attributed the change of state described by the sentence to a specific cause accessible from the contextual setting and one that excluded the existence of a concrete cause for the same change of state. The prediction derived from the general hypothesis regarding the expletiveness of anticausative voice (Alexiadou et al., 2015) was that voice morphology would not affect the interpretation. With context being the main source of relevant information, the 40 items that were introduced by an overt cause context would elicit high saliency ratings for the cause interpretation, while the 40 remaining items, introduced by a non-overt cause context, would lead to high saliency ratings for the no-cause interpretation.

The participants were given the following instructions: “In what follows you will read a set of small texts. Each text consists of the description of a situation followed by an utterance. First, we ask you to rate how natural each utterance is with respect to the situation using a scale (left edge = totally unnatural, right edge = absolutely natural). Second, we ask you to use a similar scale to rate how salient each of the two provided interpretations of the utterance is, always in relation to the situation (left edge = impossible, right edge = extremely possible).”^10^

This second experiment included the same test sentences as Experiment 1 (20 criticals +20 control items), each appearing in 2 different contextual settings. This led to a total of 80 experimental items. In order to reduce the expected duration of the task, we decided to split the items in half and create two different versions of Experiment 2. Each version included 20 critical items (5 critical verbs × 4 conditions) and 20 control items (5 control verb meanings × 4 conditions). Our participants rated all the items, producing 3 different ratings for each: one related to the naturalness of the test sentence and two related to the saliency of the two possible interpretations. A total of 9,120 responses (76 participants × (40 × 3) ratings) were statistically analyzed.

#### 2.2.3. Procedure

The procedure followed for Experiment 2 was similar to the one described for Experiment 1. Participants carried out the required tasks using their personal computers or smart devices. After reading the instructions, they filled in the same sociolinguistic questionnaire as in the first experiment (see Appendix). Then, they moved to the main part of Experiment 2. For each item, participants were presented with a context, a test sentence and two possible interpretations of the test sentence. First, they were asked to rate the naturalness of the sentence with respect to the context. Then, they were asked to decide how salient each of the interpretations was, in relation to the context-sentence pair they had to evaluate.

The items were randomized. Below we demonstrate what participants saw on their screens, using the example from (16a). Both the instructions and the example are here translated into English for the reader’s convenience.

**Table:** 

(18)	[The earthquake was very strong. The seven-storey building was deeply shaken.]		
	I	tixi	rayisan.
	the	walls	cracked.act.
	‘The walls cracked.’		
	*Rate how natural the utterance is in relation to the situation.*		
	katholou fisiko	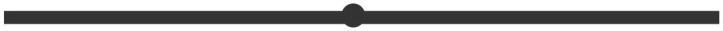	apolita fisiko
	‘totally unnatural’		‘absolutely natural’
	*Rate how salient each interpretation of the utterance is in relation to the situation.*		
	a.	An earthquake was the cause for what happened.	
	katholu pithani	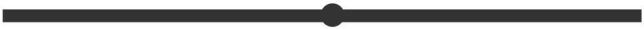	ekseretika pithani.
	‘impossible’		‘extremely possible’.
	b.	There was no specific cause for what happened.	
	katholu pithani	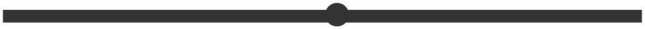	ekseretika pithani.
	‘impossible’		‘extremely possible’.

The median duration of the experiment was 18′84″.

### 2.3. Results

Due to the comparable design of the two experiments –testing the same sentences containing active and non-active anticausative pairs of the same 10 alleged Class C verbs in the absence and in the presence of context–, we report our results in a single section, divided into two parts. First, we analyze the acceptability judgments obtained *via* Experiments 1 and 2, which have been combined into a single database. The possible effect of Voice (active, non-active) is analyzed across every specific item as well as the occurrence of each item in the different contextual conditions. This analysis is run separately on control and critical items. Second, we analyze the saliency of the two possible interpretations that have been provided in Experiment 2, as reported by the participants.

Concerning the statistical analyses, a series of beta mixed-effects ANOVAs were performed using the *glmmTMB* package in R. To fulfill the requirements of a beta distribution, the 0–100 response values obtained were first divided by 100 (to obtain a 0–1 distribution), and then the two ends were replaced by very close values (0.0000001 for 0, and 0.9999999 for 1). The omnibus test results are reported, which are complemented with Sequential Bonferroni-corrected pairwise contrasts (obtained using the *emmeans* package) and Cohen’s *d* as a measure of effect size. In each reported model, the chosen random effects’ structure was the most complex structure providing no model convergence problems.

#### 2.3.1. Acceptability results

##### 2.3.1.1. Acceptability results for control items

**Figure 1:**
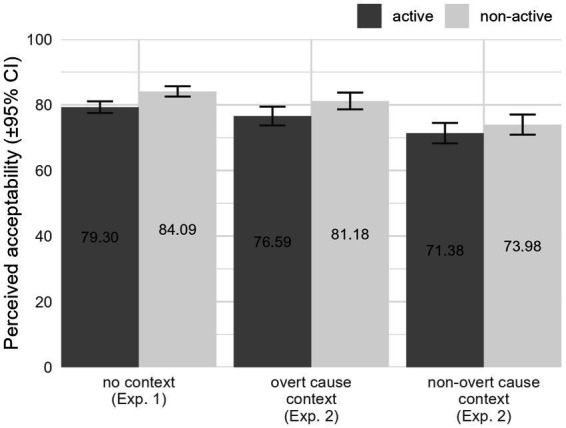
Acceptability results of control items in Experiments 1 and 2.

Figure 1 displays the results of the perceived acceptability ratings among the control items of Experiments 1 and 2. As can be seen, there is a preference for non-active voice items, a sort of preference for items presented without a context, and, among the items that did involve a context, a preference for those with an overt cause one. In general, our control items received mean acceptability ratings higher than 70%, indicating that our participants were indeed capable of providing judgments on Greek voice morphology.

A beta mixed-effects model was performed for the acceptability responses for control items of Experiments 1 and 2. The fixed factors were Voice (i.e., active, non-active), Context (i.e., no context, overt cause context, non-overt cause context) and their paired interaction. The random effects’ structure included a random intercept for Subject plus a random slope for Context by Item.

The omnibus test results showed a significant main effect for both Voice, *χ*^2^(1) = 11.676, *p* = 0.001, and Context, *χ*^2^(2) = 10.152, *p* = 0.006, but no significant interaction. The main effect of Voice relates to an overall greater acceptability of items presented with non-active morphology (*d* = 0.177, *p* = 0.001). The main effect of Context indicates lower acceptability rates for items presented with a non-overt cause context, compared to those presented with no context at all (*d* = 0.696, *p* = 0.016), and those presented with an overt cause context (*d* = 0.381, *p* = 0.039), with no significant difference between the latter (*d* = 0.315, *p* = 0.561).

An analysis of the potential effect of Voice within every specific control item was also performed. Recall that the control items used in Experiments 1 and 2 were built around 20 anticausative verbs, organized in 10 pairs of synonymous lexical items. Each pair consisted of one verb that obligatorily displayed active voice morphology (Class B) and one verb that was obligatorily marked as non-active (Class A). With this in mind, we combined the participants’ responses to both experiments into a single database and compared the ratings attributed to the two anticausative verbs within each pair of synonyms.

A model was run including Voice (active, non-active), Context (no context, overt cause context, non-overt cause context), the specific Item (pair of synonyms), and all their possible interactions as fixed factors, with a random intercept for Subject.

The three main effects and two paired interactions were found to be significant, including the double interaction Voice × Item, *χ*^2^(9) = 30.610, *p* < 0.001. This was not the case for the triple interaction, *χ*^2^(18) = 16.050, *p* = 0.589. The results of the pairwise contrasts for the Voice × Item interaction in which Voice is taken as the contrast field can be summarized in Table 1.

**Table 1:** Mean (and standard deviation) values for the reported acceptability of each specific control item across active and non-active voice in Experiments 1 and 2.

Item: Class B/Class A	Mean (SD) acceptability reported		Pairwise contrasts	
	Active voice	Non-active voice	Cohen’s *d*	Value of *p*
*alazo/metavalome* ‘change’	85.08 (20.47)	82.29 (20.98)	0.188	0.269
*salevo/trelenome* ‘go crazy’	77.83 (28.21)	**86.25 (19.71)**	−0.366	0.037
*xiroterevo/epidhinonome* ‘deteriorate’	83.93 (22.87)	89.07 (18.00)	−0.071	0.680
*meghalono/anaptisome* ‘grow’	79.41 (23.11)	81.80 (23.93)	−0.192	0.272
*lighostevo/mionome* ‘diminish’	66.41 (31.30)	67.45 (32.29)	−0.189	0.280
*skao/ekrighnime* ‘explode’	75.96 (31.57)	82.42 (26.84)	−0.174	0.347
*kaliterevo/veltionome* ‘improve’	74.78 (29.83)	**90.28 (17.21)**	−0.596	0.001
*anapodhoyirizo/anatrepome* ‘overturn’	81.97 (25.33)	87.12 (19.27)	−0.273	0.105
*yirizo/peristrefome* ‘rotate’	60.74 (34.84)	58.16 (36.16)	0.266	0.134
*vuliazo/vithizome* ‘sink’	82.89 (23.85)	**87.41 (20.54)**	−0.341	0.044

The *Active voice* column corresponds to Class B verbs, while the *Non-active voice* column corresponds to the verbs of Class A. The last two columns indicate the results of the pairwise contrasts associated with the significant interaction Voice × Item. Statistically significant results appear in bold.

As for the *non-significant* interaction Voice × Context × Item, the results of the pairwise contrasts show a scarce statistical relevance for Voice, i.e., only for three out of the thirty combinations of Item and Context. In this respect, *alazo* vs. *metavalome* ‘change’ shows a preference for active voice when no context is provided (*d* = 0.419, *p* = 0.049), *kaliterevo* vs. *veltionome* ‘improve’ shows a preference for non-active voice when no context is provided (*d* = −1.115, *p* < 0.001), and *vuliazo* vs. *vithizome* ‘sink’ shows a preference for non-active voice when a non-overt cause context is provided (*d* = −0.665, *p* = 0.047).

##### 2.3.1.2. Acceptability results for critical items

**Figure 2:**
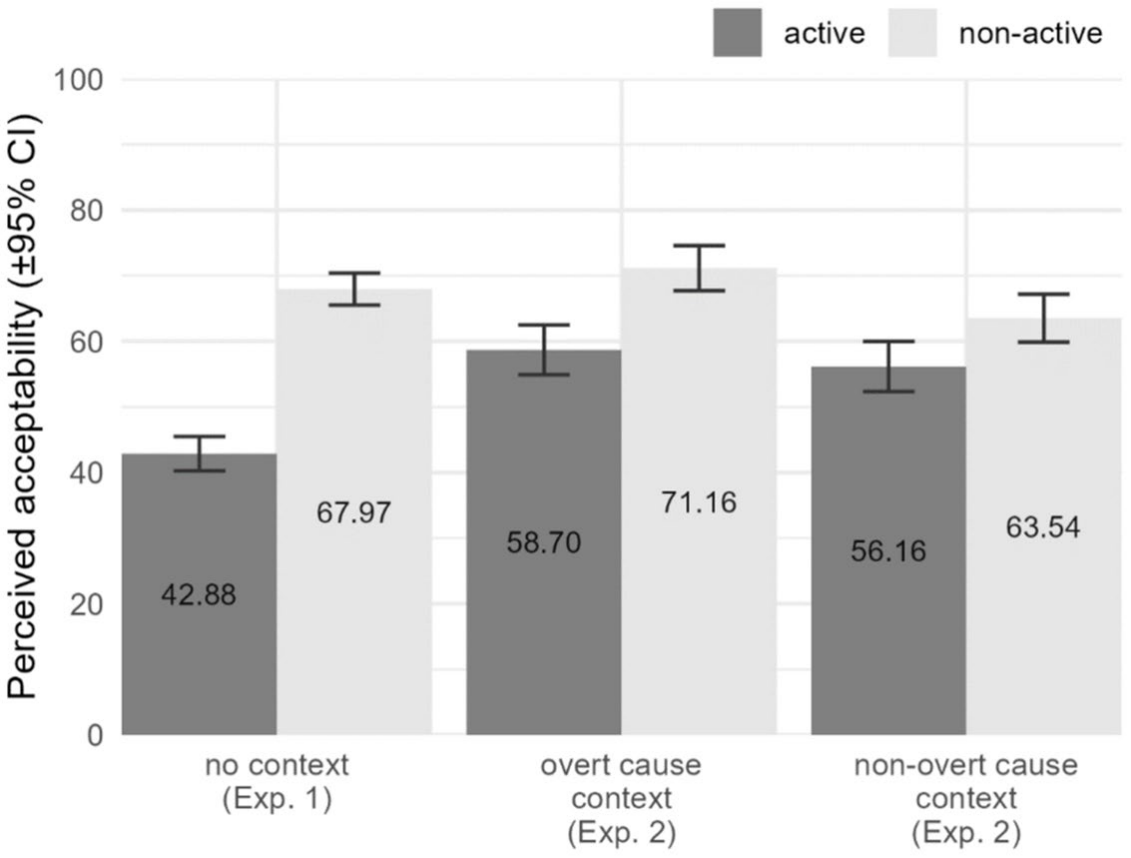
Acceptability results of critical items in Experiments 1 and 2.

Figure 2 displays the results of the perceived acceptability ratings among the critical items of Experiments 1 and 2. As can be seen, the acceptability obtained does not vary much across the different context conditions, even though a generalizable preference for non-active voice forms over active ones seems to occur. Nevertheless, the statistical results below indicate that this preference for non-active voice items is just an artifact caused by the specific verbs selected for the experimental tasks. Specifically, six out of the 10 verbs tested display a preference for non-active voice forms, three of the tested verbs display a preference for active voice forms, and one can take either the active or the non-active morphology (see Table 2 below).

**Table 2:** Mean (and standard deviation) values for the reported acceptability of each specific critical item across active and non-active voice morphology in both Experiment 1 and 2.

Item		Mean (SD) acceptability reported		Pairwise contrasts	
		Active voice	Non-active voice	Cohen’s *d*	Value of *p*
*rayizo*	‘crack’	**83.56 (23.23)**	20.84 (28.75)	4.046	<0.001
*gremizo*	‘crumble’	26.70 (32.76)	**88.24 (18.94)**	−3.489	<0.001
*tsalakono*	‘crumple’	27.02 (32.56)	**87.07 (20.36)**	−3.936	<0.001
*erimono*	‘desert’	81.73 (25.94)	76.88 (29.97)	0.286	0.283
*dhialio*	‘disperse’	32.06 (36.01)	**87.42 (20.38)**	−3.227	<0.001
*zesteno*	‘heat’	55.16 (36.83)	**77.73 (24.95)**	−1.116	<0.001
*madhao*	‘pluck’	**64.90 (34.49)**	29.89 (32.60)	2.593	<0.001
*lerono*	‘sully’	30.88 (33.46)	**79.70 (29.02)**	−3.021	<0.001
*skizo*	‘tear’	21.48 (29.77)	**87.04 (18.84)**	−3.953	<0.001
*zarono*	‘wrinkle’	**74.65 (30.42)**	38.08 (34.81)	2.465	<0.001

The last two columns indicate the results of the pairwise contrasts associated with the significant interaction Voice × Item. Statistically significant results appear in bold.

A beta mixed-effects model was performed for the acceptability responses for critical items of Experiments 1 and 2. Again, the fixed factors were Voice (i.e., active, non-active), Context (i.e., no context, overt cause context, non-overt cause context) and their paired interaction. The random effects’ structure included a random intercept for Subject plus a random slope for Voice by Item.

The omnibus test results showed significant results for Context, *χ*^2^(2) = 14.501, *p* = 0.001, and for the paired interaction Voice × Context, *χ*^2^(1) = 40.473, *p* < 0.001. However, no significant main effect was found for Voice, *χ*^2^(1) = 1.447, *p* = 0.229 (which is in line with the hypothesis that the effect of Voice is verb-specific). The main effect of Context indicates higher acceptability rates for items presented with an overt cause context, compared to those presented with no context at all (*d* = 0.605, *p* = 0.015), and those presented with a non-overt cause context (*d* = 0.564, *p* = 0.014), with no significant difference between the latter (*d* = 0.041, *p* = 1.000).

The interaction Voice × Context can be interpreted as such that different preferences for Context conditions are found when exploring active or non-active morphology. On the one hand, when active morphology is used, items without a context receive lower acceptability ratings, compared to those with overt cause contexts (*d* = 0.939, *p* < 0.001) and with non-overt cause contexts (*d* = 0.714, *p* = 0.007), with no significant difference between the latter two (*d* = 0.224, *p* = 0.620). On the other hand, when non-active morphology is used, items accompanied with a non-overt cause context receive lower acceptability ratings, compared to those with overt cause contexts (*d* = 0.572, *p* = 0.003) and those presented without a context (*d* = 0.493, *p* = 0.021), with no significant difference between the latter (*d* = 0.079, *p* = 1.000).

An additional statistical model was run over the acceptability of critical items, including Voice (active, non-active), Context (no context, overt cause context, non-overt cause context), the specific Item, and all their possible interactions as fixed factors. The model included a random slope for Voice by Subject. In this analysis, we were interested in the potential effect of Voice within every specific item and every contextual condition in which each item has been presented.

All main effects and interactions were found to be significant. The ones of interest to us are, first, the paired interaction Voice × Item, *χ*^2^(9) = 123.220, *p* < 0.001, and, second, the triple interaction Voice × Context × Item, *χ*^2^(18) = 75.480, *p* < 0.001. The pairwise contrasts associated with the paired interaction Voice × Item are summarized in Table 2: while active voice morphology is preferred for *rayizo* ‘crack’, *madhao* ‘pluck’, and *zarono* ‘wrinkle’, non-active voice is preferred for *gremizo* ‘crumble’, *tsalakono* ‘crumple’, *dhialio* ‘disperse’, *zesteno* ‘heat’, *lerono* ‘sully’, and *skizo* ‘tear’, and no significant preference is found for *erimono* ‘desert’.

Regarding the effect of Voice in the triple interaction Voice × Context × Item, it is statistically relevant only for two verbs, i.e., *erimono* ‘desert’ and *zesteno* ‘heat’. In these cases, Voice plays a role in the reported acceptability only when no contextual information is provided (i.e., only in Experiment 1), but not when there is an overt or a non-overt cause context. Specifically, *erimono* shows a significant preference for active voice (*d* = 0.720, *p* = 0.026), and *zesteno* displays a significant preference for non-active voice morphology (*d* = −2.631, *p* < 0.001).

A *post hoc* analysis was run. A single variable indicating the participants’ overall preference for active vs. non-active forms was obtained from the acceptability responses to the critical items of both Experiment 1 and 2. Using the function *partykit::ctree()*, the tested verbs were found to be best classified into two groups: Group A, featuring the verbs with high preference for active forms (*erimono* ‘desert’, *madhao* ‘pluck’, *zarono* ‘wrinkle’, *rayizo* ‘crack’), and Group B, including the verbs with low preference for active forms (*gremizo* ‘crumble’, *lerono* ‘sully’, *dhialio* ‘disperse’, *skizo* ‘tear’, *zesteno* ‘heat’, *tsalakono* ‘crumple’).

The verb classification variable, labeled as VGroup, was modeled along with Voice and Context (with all their possible interactions) in a new generalized linear mixed model. Contrasting the acceptability between the overt and non-overt cause contexts for each combination of VGroup × Voice, Group A verbs with active morphological marking (felicitous combination, mean acceptability = 0.763) showed no significant preference for any context; Group A verbs with non-active morphological marking (infelicitous, *M* = 0.419) showed a preference for overt cause contexts vs. no context (*d* = 0.764, *p* = 0.008); Group B verbs with active morphological marking (infelicitous, *M* = 0.321) showed a significant preference for context-accompanied stimuli (*d* = 1.07–1.43, *p* < 0.001); finally, Group B verbs with non-active morphological marking (felicitous, *M* = 0.846) were dispreferred when presented with a non-overt cause context (*d* = 0.75–1.07, *p* ≤ 0.002).^11^

#### 2.3.2. Interpretation results

**Figure 3:**
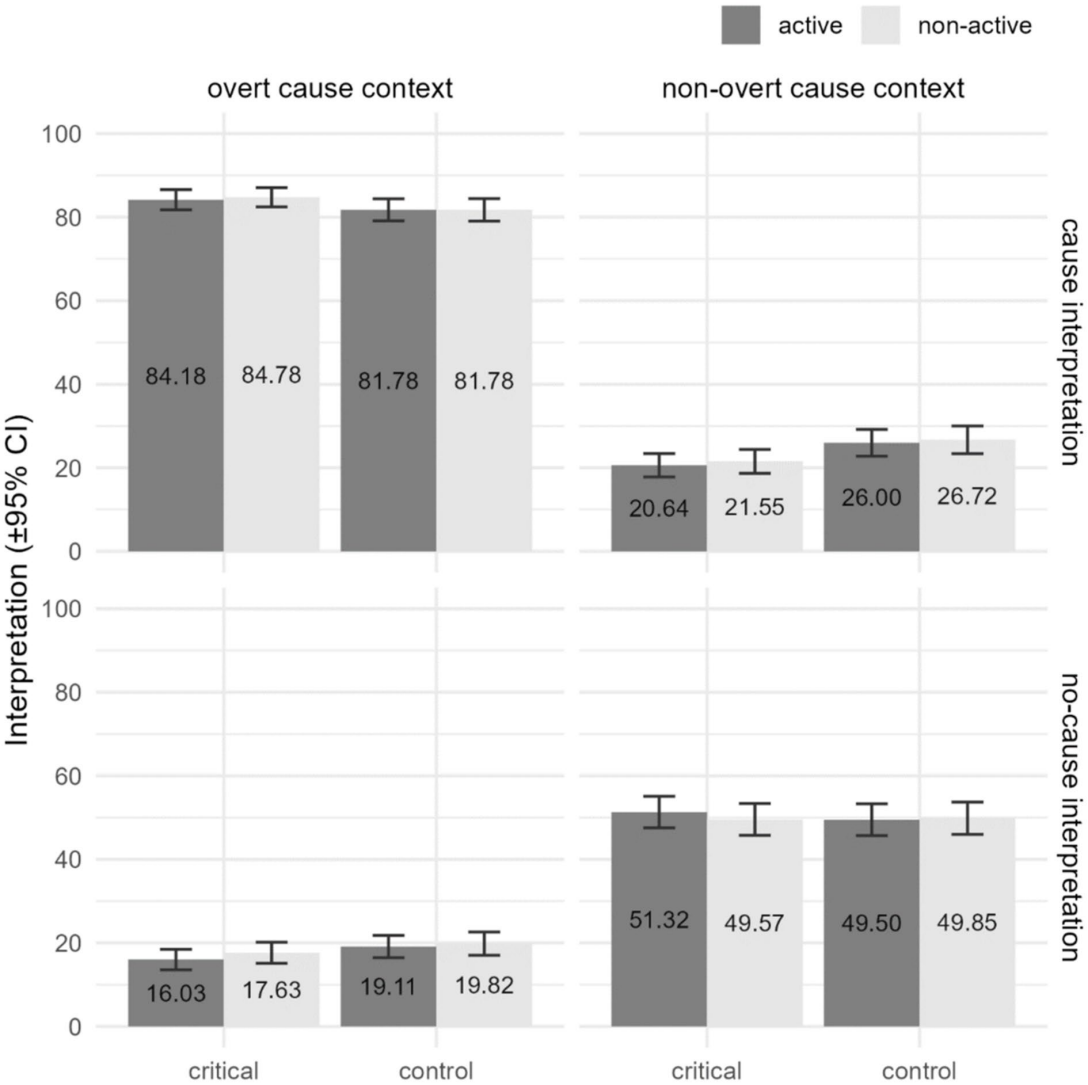
Reported saliency of the two possible interpretations offered in Experiment 2: cause and no-cause interpretations (by rows), for items presented with an overt cause context or a non-overt cause context (by columns), across Item Type and Voice.

Figure 3 illustrates how salient the participants consider the two possible interpretations offered in Experiment 2. While the first row depicts the reported saliency of a cause interpretation for items presented with an overt cause context (left) and a non-overt cause context (right), the second row shows the reported saliency of a no-cause interpretation in the same types of contexts. Overt cause contexts favor a cause interpretation and disfavor a no-cause interpretation; non-overt cause contexts disfavor a cause interpretation, while being unclear as regards no-cause interpretations.

A statistical model was run taking the reported saliency of the specific interpretation as the dependent variable. It included as fixed factors Voice (active, non-active), Context (overt cause context, non-overt cause context), Interpretation (cause interpretation, no-cause interpretation), and Item Type (critical, control). The random effects’ structure included a random slope for both Context and Interpretation by Subject, plus a random intercept for Item.

Four fixed effects were found to be significant, i.e., two main effects and two interactions. The significant main effects were Context, *χ*^2^(1) = 67.789, *p* < 0.001, and Interpretation, *χ*^2^(1) = 54.487, *p* < 0.001, though they are better explained by looking at their paired interaction, which was also found to be significant, *χ*^2^(1) = 2185.895, *p* < 0.001. The paired interaction Context × Interpretation can be explained in two complementary ways. First, cause interpretations are rated higher in overt cause contexts than in non-overt cause contexts (*d* = 4.161, *p* < 0.001), and no-cause interpretations are considered more salient in non-overt cause contexts than in overt cause contexts (*d* = −2.127, *p* < 0.001). Alternatively, for overt cause contexts, cause interpretations receive higher ratings than no-cause interpretations (*d* = 4.340, *p* < 0.001), whereas for non-overt cause contexts, no-cause interpretations receive higher ratings than cause interpretations (*d* = 1.947, *p* < 0.001). Lastly, the other interaction found to be significant was the triple interaction Context × Interpretation × Item Type, *χ*^2^(1) = 8.352, *p* = 0.004, which can be related to the fact that, in assigning a cause interpretation to a non-overt cause context, control items obtained higher ratings than critical items (*d* = 0.290, *p* = 0.013). No significant effect of Voice was found whatsoever.

Since we were interested in knowing whether there is a significant difference between the two less preferred interpretations (i.e., cause interpretation of non-overt cause contexts vs. no-cause interpretation of overt cause contexts) and between the two preferred ones (i.e., cause interpretation of overt cause contexts vs. no-cause interpretation of non-overt cause contexts), an additional statistical model was run in which the combination of Context and Interpretation was modeled as a single variable with four levels (i.e., the four panels in Figure 3). The results indicate no significant differences between the two less preferred interpretations (*d* = 0.178, *p* = 0.483), and significantly greater values for the cause interpretation of overt cause contexts compared to the no-cause interpretation of non-overt cause contexts (*d* = 1.888, *p* < 0.001). The rest of the effects described above were found intact.^12^

## 3. Discussion: The role of voice in Greek anticausatives

Let us now take stock of our findings. Experiment 1 tested the active vs. non-active voice morphology distinction in 10 Class C (Alexiadou et al., 2015) anticausative verbs. Crucially, all the verbs that formed part of our experiment were found to behave as members of either Class A (*gremizo* ‘crumble’, *tsalakono* ‘crumple’, *dhialio* ‘disperse’, *zesteno* ‘heat’, *lerono* ‘sully’, *skizo* ‘tear’) or Class B (*rayizo* ‘crack’, *erimono* ‘desert’, *madhao* ‘pluck’, *zarono* ‘wrinkle’). That is, for the majority of alternating verbs, speakers know that the anticausative variant shows a tendency toward either active voice marking or non-active voice marking. Consequently, participants systematically choose active voice morphology for some verbs and non-active voice morphology for a different group of verbs, thus indicating that Class C of Greek anticausatives is not productive (*cf.* Alexiadou et al., 2015).^13^ That said, our results further show that membership of a given anticausative verb in a morphological verb class is not categorical. In this sense, *gremizo* ‘crumble’ could be considered as a more representative member of Class A than *zesteno* ‘heat’; see Table 2.

Experiment 2 tested the same active vs. non-active voice distinction in Class C anticausatives against a contextual setting that was phrased in such a way that favored a (non-)salient cause (i.e., overt cause vs. non-overt cause context). Interestingly, with the exception of *erimono* ‘desert’ and *zesteno* ‘heat’ which showed a Class C behavior in this second experiment (no significant preference for either active or non-active voice marking), the results of Experiment 1 were replicated: *gremizo* ‘crumble’, *tsalakono* ‘crumple’, *dhialio* ‘disperse’, *lerono* ‘sully’, and *skizo* ‘tear’ behaved as Class A verbs, while *rayizo* ‘crack’, *madhao* ‘pluck’, and *zarono* ‘wrinkle’ displayed a Class B behavior. The findings of Experiment 2 suggest that the speakers’ preference for either active or non-active morphological marking in Greek anticausatives is not sensitive to the saliency of an external initiator bringing about the described change of state (*cf.* Oikonomou, 2014). Moreover, by reproducing the preference pattern obtained *via* Experiment 1, this second experiment offers additional evidence supporting the conclusion obtained from Experiment 1, namely that Class C is not productive in the grammar of native Greek speakers.

The results of Experiment 2 lead to another interesting conclusion relative to the interpretation of Greek anticausatives. Recall that, in addition to rating the naturalness of the test sentence, we asked participants to evaluate the saliency of two different interpretations at the end of each item –one that was linked to the specific cause mentioned in the contextual setting, and one that denied the existence of such a link. Irrespective of the voice morphology on the anticausative verb, participants provided low saliency ratings for those interpretations that did not match their respective context, i.e., cause interpretations under non-overt cause contexts and no cause interpretations under overt cause contexts. These ratings arguably reflect the clash between the information provided by the context and the information provided by the interpretation and, concretely, the clash between the explicit specification of an external initiator bringing about the described change of state [the earthquake in our example (16)] and the inferred absence of such an initiator for the same change of state.

On the other hand, our participants provided high saliency ratings in those cases where the information contributed by the interpretation and the context matched: cause interpretations under overt cause contexts and no cause interpretations under non-overt cause contexts. Crucially, the preference for the no cause interpretation in non-overt cause contexts was significantly lower than the preference for the cause interpretation in overt cause contexts. This result cannot be explained under the assumption that the speakers’ saliency ratings were exclusively based on the interaction between context and interpretation. Instead, it can be accounted for if a causative semantics for both active and non-active Greek anticausatives, independently argued for in the literature (Alexiadou et al., 2015), is adopted.^14^

Under the view that the saliency ratings represented in Figure 3 are based on the interaction between (i) the contextual setting, (ii) the given interpretation, and (iii) the causative semantics of anticausatives, the obtained pattern can be straightforwardly derived: The overt cause context – cause interpretation condition is the optimal condition due to the fact that the context, the interpretation, and the semantics of the anticausative included in the test sentence coincide in favoring the existence of a specific cause bringing about the described change. Therefore, in this condition we get the highest ratings. Moving to the non-overt cause context – no cause interpretation condition, this can be characterized as suboptimal; the context and the interpretation coincide in favoring the absence of a specific cause, but they both go against the causative semantics of the anticausative in the test sentence. It is this suboptimality that explains the ratings obtained. Lastly, the overt cause context – no cause interpretation and the non-overt cause context – cause interpretation conditions trigger extremely low saliency ratings due to the fact that the information clash between the context and the interpretation immediately gives rise to an incongruent output.^15^

Summing up the discussion so far, our experimental findings can be compressed into the three empirical generalizations that follow: (i) Class C of Greek anticausatives is not productive; the majority of alternating verbs falls either into Class A or Class B. (ii) The speakers’ preference for active or non-active voice marking on Greek anticausatives does not depend on the presence of some explicitly stated external initiator in the contextual setting. (iii) Native Greek speakers prefer to semantically compute/accommodate a cause when interpreting anticausative events (irrespective of the voice morphology on the verb and, partly, irrespective of the available contextual information), especially whenever the provided context and interpretation match.

The question raised next is how these generalizations relate to the theory of Greek anticausatives. In this sense, we next examine what they tell us about the hypothesis made in the literature (Alexiadou et al., 2015) according to which the VoiceP projected in anticausatives with non-active voice morphology is expletive.

Our empirical generalizations (i) and (ii) suggest that the so-called Class C is not an appropriate contrast field on which to pursue some specific meaning ascription to the voice of Greek anticausatives. Researchers attempting to do that have compared the actively and the non-actively marked anticausative variant of the same verb in different syntactic or interpretative contexts. However, we got evidence that speakers most usually prefer clearly one of the two variants. We further found that, even if they accepted both, context cannot affect or determine the choice between one variant or the other.

It seems, then, that Greek grammar gives speakers two options in forming an anticausative: either to mark the verb with active voice morphology or to mark it with non-active voice morphology. Whether an alternating verb falls in the scope of the former or the latter grammatical rule appears to be something that needs to be learnt by those acquiring/learning the language (see also Alexiadou et al., 2015).^16^ The verb-specific and, therefore, learnt status of the active and non-active voice marking of Greek anticausative verbs suggests that actively and non-actively marked anticausatives are semantically equivalent in terms of event structure. Consequently, our findings appear to be consistent with the hypothesis that the VoiceP of non-actively marked anticausatives is interpreted as introducing an identity function over predicates of events and, in this sense, can be dubbed to be expletive (Schäfer, 2008; Alexiadou et al., 2015; Oikonomou and Alexiadou, 2022).

We should note that the experimental results related to our control items can also be viewed as evidence pointing to the expletiveness direction. We remind the reader that our controls, in both experiments, involved 10 pairs of synonymous anticausative verbs, each pair consisting of one Class A verb, that was marked with non-active voice morphology, and one Class B verb, that was morphologically marked as active. When comparing the ratings given to the members of each pair (see the Results section 2.3.1.1), this synonymy was experimentally supported for 7 out of the 10 pairs tested. The very existence of synonymy across the morphologically distinct anticausative Classes A and B points toward the view that anticausative Voice does not affect the truth-conditional meaning of the sentences in which the verb appears and, thus, the expletive status of anticausative Voice are strengthened by our findings.^17^

In a recent study on expletiveness across several functional categories, Tsiakmakis and Espinal (2022) reach the following generalization: semantically expletive categories, apart from introducing an identity function at the truth-conditional level, need to stand in a local syntactic relation to an element with respect to which they initially encode some redundant meaning. If Voice in Greek non-actively marked anticausatives is interpreted as expletive, it is expected to satisfy this precondition. We claim that this prediction is borne out, and that this is exactly where our empirical generalization (iii), namely that speakers prefer to interpret a cause when faced with anticausative structures, becomes relevant.^18^

Let us adopt for our discussion the main aspects of the syntactic account by Alexiadou et al. (2015). In the authors’ view, anticausatives correspond primarily to causative little *v*Ps, as represented in (19b); those that are marked with non-active voice morphology further project a non-active VoiceP lacking a specifier above *v*P, as shown in (20b).

**Table:** 

(19)	a.	I	fusta	zarose.
		the	skirt	wrinkled.act.
		‘The skirt wrinkled.’		
	b.	[_vP_ [_v_ v √zaron] [_DP_ i fusta]]		
(20)	a.	I	thalasa	zesta*thik*e.
		the	sea	heated.nact
		‘The sea heated.’		
	b.	[_VoiceP-NACT_ [_Voice-NACT_ thik-] [_vP_ [_v_ v √zesten] [_DP_ i thalassa]]]		

As regards the semantics attributed to such structures, it is postulated that, up to the *v*P level in both actively and non-actively marked anticausatives, the interpretation is one of a *causative* event, because *v* has a causative flavor. Crucially, this is in accordance with our experimental finding that, regardless of voice morphology, speakers interpret/accommodate a cause for anticausatives (see Figure 3). The question that is at the heart of the present theoretical analysis is in what way the projection of the non-active VoiceP in (20b) contributes compositionally to the meaning of the sentence.We postulate that the non-active Voice that emerges as non-active morphological marking in Greek anticausatives encodes a cause-related meaning, i.e., that it is initially non-expletive. On this basic tenet, we coincide with Oikonomou (2014). This non-active Voice head merges with a *v*P that also involves a cause formal feature. The resulting redundancy of cause-related information in the structure –that is, the multiple representation of some cause semantic feature within a syntactically local domain (see Tsiakmakis and Espinal, 2022)– leads to the interpretation of this Voice as an identity function over the predicate of events introduced by the causative *v*.^19^ In other words, the expletive interpretation of Voice in anticausatives is a by-product of it merging with a causative *v*.

Following this approach, Voice in Greek anticausatives ultimately introduces no semantic argument; it ends up being interpreted as an identity function, as a result of an emerging semantic redundancy. In this way, we correctly predict that there is no traceable truth-conditional asymmetry whatsoever between an actively and a non-actively marked anticausative, that is between a Voice-less anticausative and an anticausative projecting a non-active VoiceP. The meaning of Greek anticausative Voice is represented formally in (21) (Schäfer, 2008, 2017; Wood, 2014, 2015; Schäfer and Vivanco, 2016), where 〈s,t〉 stands for the situation (or event) in which the proposition is true. The partial semantics for (19) and (20) is given in (22) and (23), respectively.

**Table:** 

(21)	Anticausative Voice: ⟦*-thik-*_CAUSE_⟧^20^ = λP_〈s,t〉_.P_〈s,t〉_
(22)	⟦[_v_ cause [_VP_ *I fusta zaron*]]⟧ = λe_CAUS_ [wrinkle(e) & theme(e) = the skirt]
(23)	⟦[_Voice_ *-thik-*_CAUSE_ [_v_ cause [_VP_ *I thalasa zesten*]]]⟧
	= (λP_〈s,t〉_.P_〈s,t〉_) (λe_CAUS_ [heat(e) & theme(e) = the sea])
	= λe_CAUS_ [heat(e) & theme(e) = the sea]

As stated throughout the paper, the idea that non-actively marked anticausatives project an expletive Voice is not new; it is present in Schäfer (2008) and Wood (2014) and is put forth explicitly in relation to Greek in Alexiadou et al. (2015). The finer insight that this expletiveness is dependent on the syntactic context is not new either; see Oikonomou and Alexiadou (2022). What is novel in the present proposal is that the expletiveness of Voice is legitimized by its local relation to the causative *v*, not by unspecified information attributed to the verbal root (*cf.* Alexiadou et al., 2015; Oikonomou and Alexiadou, 2022).^21^ This departure from previous approaches is not trivial and is motivated by the inclusion of Greek anticausative Voice in the typology of semantic expletives, which also include expletive negation and expletive plural number on mass nouns (see Tsiakmakis and Espinal, 2022).^22^

## 4. Conclusion

In this paper we investigated voice in Greek anticausatives. We ran an experimental study that explored Greek native speakers’ preferences with respect to two different judgment tasks that aimed to shed light on the distribution and interpretation of anticausative voice in Greek, a topic clouded by a heated debate.

The results of our experiments support the following conclusions: (i) The so-called Class C of Greek anticausative verbs is not productive; for the majority of alternating verbs, speakers have a clear preference for either the anticausative variant that bears active voice morphology (Class B) or the variant that displays non-active voice morphology (Class A). (ii) The speakers’ preference for either form of the anticausative verb is not significantly affected or determined by the presence/absence of a linguistically phrased external initiator bringing about the described change of state. (iii) Regardless of voice morphology, speakers tend to interpret/accommodate a cause when faced with anticausative event descriptions, provided that the information surrounding the event description is not contradictory (see Graphs 1 and 4 in Figure 3).

Our first and second empirical generalizations suggest that the emergence of active or non-active voice morphology in Greek anticausatives is verb-specific and, thus, encoded in the lexicon. Furthermore, these generalizations can be regarded as evidence consistent with the theoretical proposal that the non-active Voice that is realized as non-active morphology in Greek anticausatives is expletive, interpreted as introducing an identity function over predicates of events (Schäfer, 2008, 2017; Wood, 2014, 2015; Alexiadou et al., 2015). Taking into account also the third empirical generalization, we can further refine this proposal: in Greek anticausatives, a non-expletive Voice head carrying cause-related information merges with a causative little *v.* The resulting redundancy of cause information allows the Voice of Greek anticausatives to behave as a typical semantically expletive category (Tsiakmakis and Espinal, 2022): it stands in a local relationship with an element that also encodes causative meaning and is, ultimately, interpreted merely as an identity function over causative events.

In light of the above, the present study can be regarded as a solid source of both empirical and theoretical evidence in support of the expletiveness hypothesis regarding the voice of Greek anticausatives. While extending our view to other languages requires further research, a paradigm has been set to pursue relevant queries across languages.

## Data availability statement

The raw data supporting the conclusions of this article will be made available by the authors, without undue reservation.

## Ethics statement

The studies involving human participants were reviewed and approved by the Ethics Committee on Animal and Human Experimentation of the Universitat Autònoma de Barcelona, under the approved experimental protocol number CEEAH-4442. The patients/participants provided their written informed consent to participate in this study.

## Author contributions

ET ran the bibliographical search and designed the materials for the experiments. ET, JB-C and ME designed the experiments. JB-C is responsible for the statistical analysis of the results. ET and ME are responsible for the whole research. All authors contributed to the article and approved the submitted version.

## Funding

This research was supported by the Spanish Ministerio de Ciencia e Innovación (FFI2017-82547-P, PID2020-112801GB-I00) and the Generalitat de Catalunya (2021SGR00787).

## Conflict of interest

The authors declare that the research was conducted in the absence of any commercial or financial relationships that could be construed as a potential conflict of interest.

## Publisher’s note

All claims expressed in this article are solely those of the authors and do not necessarily represent those of their affiliated organizations, or those of the publisher, the editors and the reviewers. Any product that may be evaluated in this article, or claim that may be made by its manufacturer, is not guaranteed or endorsed by the publisher.

## Footnotes

^1^In the rest of the paper, the word *marked* refers exclusively to the presence of either active voice morphological marking or non-active voice morphological marking.

^2^We use lowercase *voice* to refer to the corresponding morphological category and uppercase *Voice* to refer to the corresponding syntactic category.

^3^The co-existence of morphologically distinct classes of anticausative verbs within the same language is a typologically frequent situation; see Haspelmath (1993, 2016).

^4^In the rest of the paper, we gloss only the morphological distinctions that are relevant to our discussion, namely active vs. non-active voice.

^5^We have replaced Oikonomou’s (2014) question mark with the # symbol.

^6^See Lavidas et al. (2012) for a view similar in essence, according to which non-active voice morphology in Greek alternating verbs correlates with the existence of an implicit external argument.

^7^Non-actively marked anticausatives have been related also to reflexivity, especially with reference to Romance languages (Kallulli, 2006; Labelle, 2008; Koontz-Garboden, 2009, among many others). This alternative is left aside because it has not been proposed, to our knowledge, in relation to Greek. See Horvath and Siloni (2011), Alexiadou et al. (2015), Schäfer and Vivanco (2016), for arguments against the reflexivity analysis of anticausatives.

^8^We acknowledge that the speakers’ ratings of the test sentences may be affected by factors other than their preference for active or non-active morphological marking on a given verb, such as the type of the internal argument. However, upon designing the experimental study, we chose a limited set of experimental conditions.

^9^In the case of non-overt cause contexts, there was still the possibility that participants accommodated some cause for the described change of state, although one of a different type than the external initiator introduced explicitly by overt cause contexts.

^10^The reader may notice at this point that the instructions to the two experiments differed. The sentences in Experiment 1 appeared in isolation and participants had to rate them on a “bad-to-good” scale. The sentences in Experiment 2, on the other hand, were preceded by specific contextual information and participants had to judge them on a “totally unnatural-to-absolutely natural” scale with respect to the preceding context. Given that the results obtained from the two experiments showed mostly a similar behavior when looking at the item-specific variation, especially in the case of critical items, and in order to simplify the exposition, the results of the two experiments are presented jointly. Concretely, in the description of the results, we use *acceptability* as a superordinate term that includes both the *good* vs. *bad* ratings and the *natural* vs. *unnatural* ratings elicited from the participants.

^11^The reviewers soundly raise questions regarding potential inter-speaker variation. We report that the tendencies described above were found to be consistent across subjects, with only *zarono* ‘wrinkle’ and *dhialio* ‘disperse’ showing some variability.

^12^Four separate one-sample *t*-tests were performed to the four different combinations of Context × Interpretation. The category of data pertaining to the non-overt cause context × no cause interpretation condition shows a bimodal distribution, with a set of responses close to 0 and another set close to 100, which cannot be explained by means of the variable Voice. To explain some of this variation, several conditional classification trees were run. Initially, it was observed that the data was better classified into multiple groups by considering the specific verb used; in this regard, *lerono* ‘sully’, *peristrefome/yirizo* ‘rotate’, *mionome/lighostevo* ‘diminish’, *madhao* ‘pluck’, *tsalakono* ‘crumple’, and *veltionome/kaliterevo* ‘improve’ were grouped for being classified as having a no cause interpretation, in contrast with the rest of the verbs. If we disregard the effect of the specific verb, two different demographic variables allow to explain part of the variation as well. Specifically, subjects with linguistics-related studies and subjects with a postgraduate or a doctoral degree tended to accept more a no cause interpretation for the anticausatives that were used under a non-overt cause context (compared with those participants with a tertiary level of education not related to linguistics).

^13^See also Fotiadou (2022), for indirect evidence in this direction.

^14^See Koontz-Garboden (2009) for the same claim regarding languages other than Greek. Note that Alexiadou et al. (2015) argue in favor of a causative semantics for anticausatives based on the fact that the latter are compatible with modification by Prepositional Phrases carrying causer information:

**Table:**

(i)	To	spiti	gremis*tike*	*apo*	*to*	*sismo.*
	the	house	crumbled.nact	from	the	earthquake
	‘The house crumbled from the earthquake.’					

^15^A reviewer suggests that the interpretation results of Experiment 2 are compatible with an alternative view, according to which the causative meaning component of Greek anticausatives is an implicature. However, if it were indeed an implicature, the causative component would be easily canceled by both the context and the offered interpretation in the non-overt cause context – no cause interpretation condition, leading to saliency ratings as high as in the overt-cause context – cause interpretation condition. This is not what we find. The implicature alternative cannot account for the significant difference between the ratings in the optimal condition and the ratings in the suboptimal condition. As regards specifically the suboptimal condition, another reviewer suggests that the obtained results may also be attributable to the participants’ uncertainty, or the clash between a cause accommodated *via* the context and the absence of a specific cause imposed by the provided interpretation.

^16^For the very few verbs that alternate freely between non-active and active voice morphology, children acquiring Greek will also have to learn the double morphology as part of the respective lexical entries. We note that, to our knowledge, there is no acquisition study focusing exclusively on the anticausative readings of Class C verbs. The reader is referred to Fotiadou and Tsimpli (2010) for an experimental study on the acquisition of transitivity alternations in Greek.

^17^See Schäfer and Vivanco (2016) for similar argumentation in relation to languages other than Greek.

^18^Tsiakmakis and Espinal (2022) take the allegedly expletive voice marker *se* in Romance anticausatives to be a syntactically expletive category, not a semantically expletive one. However, these authors do not take into account Greek expletive voice, which arguably has different characteristics.

^19^Notice that an explanation of the expletiveness of Voice in this spirit creates the expectation that a Voice head carrying agent information can also end up being interpreted as an identity function, if merged in a structure introducing the agent elsewhere. We speculate that this is what happens in Greek deponent verbs (verbs with non-active voice morphology but transitive syntax; see Zombolou and Alexiadou, 2014 for a finer categorization), which have been argued to introduce agents at the specifier of a functional projection below VoiceP (Grestenberger, 2018). We provide an example and its partial derivation, in the spirit of such an analysis:

**Table:**

(i)	a.	I	Maria	iperaspis*tike*	ton	mikro	tis	adherfo.
		the	Maria	defended.nact	the	small	her	brother
		‘Maria defended her younger brother.’						
	b.	[_VoiceP-NACT_ [_Voice-NACT_ tik-] [_XP_ [_DP_ i Maria] [_Xˈ_ [X] [[_vP_ [_v_ v √iperaspiz] [_DP_ ton mikro tis adherfo]]]]]]						

^20^We have chosen *-thik-* as the morphological exponent of anticausative Voice in our definition because it is the one that appears in our examples. For discussion on the full paradigm of non-active voice morphology, the reader is referred to Holton et al. (1997), Ralli (2005), and Manzini et al. (2015).

^21^It should be clarified at this point that, of all the possible interpretations of Voice in Greek, our analysis aims to account only for the interpretation of the causative Voice projected in non-actively marked anticausatives as an expletive, which we consider as the result of a cause-related information redundancy. The interpretation of Greek Voice in the rest of its instances is beyond the present experimental study.

^22^Let us try to demonstrate briefly the parallel between the analysis proposed here for the expletiveness of Greek anticausative Voice and Tsiakmakis and Espinal’s (2022) account of the expletiveness of Catalan negation, with the help of the following example:

**Table:**

(i)	*Tinc*	*por*	que	*no*	es	mengin	el	pastís.
	I.have	fear	that	not	cl	eat.subj	the	cake
	‘I am afraid that they might eat the cake.’/‘I am afraid that they might not eat the cake.’							Following the authors, in this case there is a negative semantic feature in the fear predicate *tinc por* (Espinal, 1992) and another negative semantic feature in the negative marker *no*. When the complement clause is in subjunctive and, therefore, transparent to the matrix fear predicate (Picallo, 1985), the two categories carrying a negative feature are in a local syntactic relationship and semantic redundancy of negative meaning arises. This semantic redundancy opens the possibility that the negative marker *no* is interpreted merely as an identity function, giving rise to the interpretation ‘I am afraid they will eat the cake.’ (see Tsiakmakis and Espinal, 2022, for details).
