# Supplementary material for: On the distribution and interpretation of voice in Greek anticausatives
Source: Front Psychol. 2023 Feb 23;14:1068058. doi: 10.3389/fpsyg.2023.1068058 (PMC9997674; doi:10.3389/fpsyg.2023.1068058)
Supplement: Supplementary file 1 [file Data_Sheet_1.pdf]

Appendix

1. Sociolinguistic questionnaire, with English translations.

1) Ημερομηνία γέννησης Date of birth
2) Φύλο (άνδρας, γυναίκα, άλλο) Gender (male, female, other)
3) Εκπαίδευση (πρωτοβάθμια, δευτεροβάθμια, τριτοβάθμια, κάτοχος μεταπτυχιακού τίτλου, κάτοχος διδακτορικού τίτλου) Education (primary school, high school, bachelor's degree, master's degree, PhD degree)
4) Τόπος διαμονής κατά το μεγαλύτερο μέρος της παιδικής ηλικίας Place of residence during the biggest part of childhood
5) Τόπος μόνιμης κατοικίας Place of permanent residence
6) Είσαι απόφοιτος-η ή φοιτητής-τρια γλωσσολογίας ή σχετικού τμήματος; (ναι, όχι) Are you a graduate or undergraduate student of linguistics or other relevant discipline? (yes, no)
7) Είναι η ελληνική η μητρική σου γλώσσα; (ναι, όχι) Is Greek your native language? (yes, no)
8) Σε τι ποσοστό χρησιμοποιείς την ελληνική γλώσσα καθημερινά; (λιγότερο από 50%, 50% - 75%, περισσότερο από 75%) What is the percentage of your daily use of Greek? (less than 50%, 50% - 75%, more than 75%)
9) Πόσο σίγουρος-η αισθάνεσαι για την ικανότητά σου στη χρήση της ελληνικής; (λιγότερο από 50%, 50% - 75%, περισσότερο από 75%) How confident are you about your command of the Greek language? (less than 50%, 50% - 75%, more than 75%)

2. Sociolinguistic information regarding the participants of Experiments 1 and 2.

		Exp 1		Exp 2	
Participants analyzed		90		76	
Median time to complete the experiment		6.93 mins.		18.84 mins.	
Age in years (M, SD)		28.91 (3.99)		29.91 (5.69)	
		n	*%*	*n*	*%*
Gender	Male	44	48.89	20	26.32
	Female	44	48.89	55	72.37
	Other / NA	2	2.22	1	1.32
Educational level	Primary	0	0.00	0	0.00
	Secondary	6	6.67	3	3.95
	Tertiary	38	42.22	29	38.16
	Postgraduate	45	50.00	43	56.58
	Doctorate	1	1.11	1	1.32
Had studied linguistics, language, or translation	Yes	30	33.33	39	51.32
	No	60	66.67	37	48.68
Daily use of Greek	>75%	77	85.56	66	86.84
	50-75%	10	11.11	9	11.84
	<50%	3	3.33	1	1.32
Self-assessment in Greek	>75%	72	80.00	65	85.53
	50-75%	17	18.89	11	14.47
	<50%	1	1.11	0	0.00

3. Materials used in Experiment 1, with English translations.

Critical items

a. *gremizo* ‘crumble’

1 Το παλιό σπίτι της γιαγιάς γκρέμισε.

2 Το παλιό σπίτι της γιαγιάς γκρεμίστηκε.

‘Grandma’s old house crumbled.’

b. *lerono* ‘sully’

3 Οι κουρτίνες της μαμάς λέρωσαν.

4 Οι κουρτίνες της μαμάς λερώθηκαν.

‘Mum’s curtains sullied.’

c. *erimono* ‘desert’

5 Το χωριό ερήμωσε.

6 Το χωριό ερημώθηκε.

‘The village got deserted.’

d. *dhialio* ‘disperse’

7 Το πλήθος διέλυσε.

8 Το πλήθος διαλύθηκε.

‘The crowd dispersed.’

e. *skizo* ‘tear’

9 Το παντελόνι έσκισε.

10 Το παντελόνι σκίστηκε.

‘The pants teared.’

f. *madhao* ‘pluck’

11 Ο άσπρος σκύλος μάδησε.

12 Ο άσπρος σκύλος μαδήθηκε.

‘The white dog plucked.’

g. *zarono* ‘wrinkle’

13 Το μέτωπό του ζάρωσε.

14 Το μέτωπό του ζαρώθηκε.

‘His forehead wrinkled.’

h. *rayizo* ‘crack’

15 Οι τοίχοι ράγισαν.

16 Οι τοίχοι ραγίστηκαν.

‘The walls cracked.’

i. *zesteno* ‘heat’

17 Η θάλασσα ζέστανε.

18 Η θάλασσα ζεστάθηκε.

‘The sea heated.’

j. *tsalakono* ‘crumple’

19 Το εξώφυλλο τσαλάκωσε.

20 Το εξώφυλλο τσαλακώθηκε.

‘The cover crumpled.’

Control items

a. change

21 Το πολιτικό σκηνικό άλλαξε.

22 Το πολιτικό σκηνικό μεταβλήθηκε.

‘The political stage changed.’

b. sink

23 Το καράβι βούλιαξε.

24 Το καράβι βυθίστηκε.

‘The ship sank.’

c. turn over

25 Το όχημα αναποδογύρισε.

26 Το όχημα ανατράπηκε.

‘The vehicle turned over.’

d. rotate

27 Η Γη γύρισε μία φορά γύρω από τον Ήλιο.

28 Η Γη περιστράφηκε μία φορά γύρω από τον Ήλιο.

‘The Earth rotated once around the Sun.’

e. diminish

29 Ο χρόνος που του απέμενε λιγόστεψε.

30 Ο χρόνος που του απέμενε μειώθηκε.

‘The time he had left diminished.’

f. deteriorate

31 Η υγεία της χειροτέρεψε.

32 Η υγεία της επιδεινώθηκε.

‘Her health deteriorated.’

g. improve

33 Η όρασή του καλυτέρεψε.

34 Η όρασή του βελτιώθηκε.

‘His vision improved.’

h. grow

35 Ο τρυφερός βλαστός μεγάλωσε.

36 Ο τρυφερός βλαστός αναπτύχθηκε.

‘The young stem grew.’

i. go crazy

37 Οι χωριανοί έλεγαν ότι σάλεψε.

38 Οι χωριανοί έλεγαν ότι τρελάθηκε.

‘The villagers thought he went crazy.’

j. explode

39 Η βομβα έσκασε.

40 Η βόμβα εξερράγη.

‘The bomb exploded.’

4. Materials used in Experiment 2, with English translations.

Critical items

a. *gremizo* ‘crumble’

1 Active voice – Overt cause context

[Πήρε τηλέφωνο η θεία να μας πει για το σεισμό στο χωριό σήμερα το πρωί. Κουνήθηκαν πολύ.]

‘Auntie called to tell us about the earthquake at the village this morning. They shook a lot.’

Το παλιό σπίτι της γιαγιάς γκρέμισε.

‘Grandma’s old house crumbled.’

α. Ένας σεισμός ήταν η αιτία για αυτό που συνέβη. *Cause int.*

‘An earthquake was the cause of what happened.’

β. Δεν υπήρχε συγκεκριμένη αιτία για αυτό που συνέβη. *No cause int.*

‘There was no specific cause for what happened.’

2 Active voice – Non-overt cause context

[Πήγα ξανά στο χωριό μετά από πολύ καιρό. Έπρεπε να είχαμε κάνει επισκευές τόσα χρόνια.]

‘I went back to the village after a long time. We should have made repairs all these years.’

Το παλιό σπίτι της γιαγιάς γκρέμισε.

‘Grandma’s old house crumbled.’

α. Ένας σεισμός ήταν η αιτία για αυτό που συνέβη. *Cause int.*

‘An earthquake was the cause of what happened.’

β. Δεν υπήρχε συγκεκριμένη αιτία για αυτό που συνέβη. *No cause int.*

‘There was no specific cause for what happened.’

3 Non-active voice – Overt cause context

[Πήρε τηλέφωνο η θεία να μας πει για το σεισμό στο χωριό σήμερα το πρωί. Κουνήθηκαν πολύ.]

‘Auntie called to tell us about the earthquake at the village this morning. They shook a lot.’

Το παλιό σπίτι της γιαγιάς γκρεμίστηκε.

‘Grandma’s old house crumbled.’

α. Ένας σεισμός ήταν η αιτία για αυτό που συνέβη. *Cause int.*

‘An earthquake was the cause of what happened.’

β. Δεν υπήρχε συγκεκριμένη αιτία για αυτό που συνέβη. *No cause int.*

‘There was no specific cause for what happened.’

4 Non-active voice – Non-overt cause context

[Πήγα ξανά στο χωριό μετά από πολύ καιρό. Έπρεπε να είχαμε κάνει επισκευές τόσα χρόνια.]

‘I went back to the village after a long time. We should have made repairs all these years.’

Το παλιό σπίτι της γιαγιάς γκρεμίστηκε.

‘Grandma’s old house crumbled.’

α. Ένας σεισμός ήταν η αιτία για αυτό που συνέβη. *Cause int.*

‘An earthquake was the cause of what happened.’

β. Δεν υπήρχε συγκεκριμένη αιτία για αυτό που συνέβη. *No cause int.*

‘There was no specific cause for what happened.’

b. *lerono* ‘sully’

5 Active voice – Overt cause context

[Άπλωσα έξω τα λευκά να στεγνώσουν χωρίς να κοιτάξω τον καιρό. Μετά από λίγο έπιασε φοβερή βροχή.]

‘I hung the clothes out to dry without checking the weather. It started raining heavily after a while.’

Οι κουρτίνες της μαμάς λέρωσαν.

‘Mum’s curtains sullied.’

α. Η βροχή ήταν η αιτία για αυτό που συνέβη. *Cause int.*

‘The rain was the cause of what happened.’

β. Δεν υπήρχε συγκεκριμένη αιτία για αυτό που συνέβη. *No cause int.*

‘There was no specific cause for what happened.’

6 Active voice – Non-overt cause context

[Έχουμε δύο μήνες να κάνουμε γενική καθαριότητα, ήρθε η ώρα. Θα ξεκινήσω από την κουζίνα.]

‘We haven’t cleaned the house for two months, it’s time. I will start from the kitchen.’

Οι κουρτίνες της μαμάς λέρωσαν.

‘Mum’s curtains sullied.’

α. Η βροχή ήταν η αιτία για αυτό που συνέβη. *Cause int.*

‘The rain was the cause of what happened.’

β. Δεν υπήρχε συγκεκριμένη αιτία για αυτό που συνέβη. *No cause int.*

‘There was no specific cause for what happened.’

7 Non-active voice – Overt cause context

[Άπλωσα έξω τα λευκά να στεγνώσουν χωρίς να κοιτάξω τον καιρό. Μετά από λίγο έπιασε φοβερή βροχή.]

‘I hung the clothes out to dry without checking the weather. It started raining heavily after a while.’

Οι κουρτίνες της μαμάς λερώθηκαν.

‘Mum’s curtains sullied.’

α. Η βροχή ήταν η αιτία για αυτό που συνέβη. *Cause int.*

‘The rain was the cause of what happened.’

β. Δεν υπήρχε συγκεκριμένη αιτία για αυτό που συνέβη. *No cause int.*

‘There was no specific cause for what happened.’

8 Non-active voice – Non-overt cause context

[Έχουμε δύο μήνες να κάνουμε γενική καθαριότητα, ήρθε η ώρα. Θα ξεκινήσω από την κουζίνα.]

'We haven't cleaned the house for two months, it's time. I will start from the kitchen.'

Οι κουρτίνες της μαμάς λερώθηκαν.

'Mum's curtains sullied.'

α. Η βροχή ήταν η αιτία για αυτό που συνέβη. *Cause int.*

'The rain was the cause of what happened.'

β. Δεν υπήρχε συγκεκριμένη αιτία για αυτό που συνέβη. *No cause int.*

'There was no specific cause for what happened.'

<u>c. *erimono* 'desert'</u>

9 Active voice – Overt cause context

[Η πυρκαγιά που ξέσπασε ήταν καταστροφική. Τα περισσότερα σπίτια δεν ήταν πια κατοικήσιμα.]

'The fire that broke out was destructive. Most of the houses were now uninhabitable.'

Το χωριό ερήμωσε.

'The village got deserted.'

α. Μια πυρκαγιά ήταν η αιτία για αυτό που συνέβη. *Cause int.*

'A fire was the cause of what happened.'

β. Δεν υπήρχε συγκεκριμένη αιτία για αυτό που συνέβη. *No cause int.*

'There was no specific cause for what happened.'

10 Active voice – Non-overt cause context

[Καθώς περνούσε ο καιρός, οι νέοι έβλεπαν τις επιλογές τους να λιγοστεύουν. Η ζωή στην πόλη πρόσφερε περισσότερες ευκαιρίες.]

'As the years passed, young people were left no choice. Life in the city looked more promising.'

Το χωριό ερήμωσε.

'The village got deserted.'

α. Μια πυρκαγιά ήταν η αιτία για αυτό που συνέβη. *Cause int.*

'A fire was the cause of what happened.'

β. Δεν υπήρχε συγκεκριμένη αιτία για αυτό που συνέβη. *No cause int.*

'There was no specific cause for what happened.'

11 Non-active voice – Overt cause context

[Η πυρκαγιά που ξέσπασε ήταν καταστροφική. Τα περισσότερα σπίτια δεν ήταν πια κατοικήσιμα.]

'The fire that broke out was destructive. Most of the houses were now uninhabitable.'

Το χωριό ερημώθηκε.

'The village got deserted.'

α. Μια πυρκαγιά ήταν η αιτία για αυτό που συνέβη. *Cause int.*

'A fire was the cause of what happened.'

β. Δεν υπήρχε συγκεκριμένη αιτία για αυτό που συνέβη. *No cause int.*

'There was no specific cause for what happened.'

12 Non-active voice – Non-overt cause context

[Καθώς περνούσε ο καιρός, οι νέοι έβλεπαν τις επιλογές τους να λιγοστεύουν. Η ζωή στην πόλη πρόσφερε περισσότερες ευκαιρίες.]

'As the years passed, young people were left no choice. Life in the city looked more promising.'

Το χωριό ερημώθηκε.

'The village got deserted.'

α. Μια πυρκαγιά ήταν η αιτία για αυτό που συνέβη. *Cause int.*

'A fire was the cause of what happened.'

β. Δεν υπήρχε συγκεκριμένη αιτία για αυτό που συνέβη. *No cause int.*

'There was no specific cause for what happened.'

d. *dhialio* 'disperse'

13 Active voice – Overt cause context

[Χιλιάδες διαδηλωτές συγκεντρώθηκαν το πρωί στην πλατεία Αριστοτέλους. Μετά από λίγα λεπτά ξέσπασε τρομερή μπόρα.]

'Thousands of protestors gathered this morning at Aristotelous square. After some minutes, a terrible storm broke out.'

Το πλήθος διέλυσε.

'The crowd dispersed.'

α. Μια μπόρα ήταν η αιτία για αυτό που συνέβη. *Cause int.*

'A storm was the cause of what happened.'

β. Δεν υπήρχε συγκεκριμένη αιτία για αυτό που συνέβη. *No cause int.*

'There was no specific cause for what happened.'

14 Active voice – Non-overt cause context

[Ο εορτασμός της επετείου στέφθηκε με επιτυχία. Η παρέλαση ολοκληρώθηκε περίπου στις 12:00.]

'The celebration of the anniversary was successful. The parade finished at around 12:00.'

Το πλήθος διέλυσε.

'The crowd dispersed.'

α. Μια μπόρα ήταν η αιτία για αυτό που συνέβη. *Cause int.*

'A storm was the cause of what happened.'

β. Δεν υπήρχε συγκεκριμένη αιτία για αυτό που συνέβη. *No cause int.*

‘There was no specific cause for what happened.’

15 Non-active voice – Overt cause context

[Χιλιάδες διαδηλωτές συγκεντρώθηκαν το πρωί στην πλατεία Αριστοτέλους. Μετά από λίγα λεπτά ξέσπασε τρομερή μπόρα.]

‘Thousands of protestors gathered this morning at Aristotelous square. After some minutes, a terrible storm broke out.’

Το πλήθος διαλύθηκε.

‘The crowd dispersed.’

α. Μια μπόρα ήταν η αιτία για αυτό που συνέβη. *Cause int.*

‘A storm was the cause of what happened.’

β. Δεν υπήρχε συγκεκριμένη αιτία για αυτό που συνέβη. *No cause int.*

‘There was no specific cause for what happened.’

16 Non-active voice – Non-overt cause context

[Ο εορτασμός της επετείου στέφθηκε με επιτυχία. Η παρέλαση ολοκληρώθηκε περίπου στις 12:00.]

‘The celebration of the anniversary was successful. The parade finished at around 12:00.’

Το πλήθος διαλύθηκε.

‘The crowd dispersed.’

α. Μια μπόρα ήταν η αιτία για αυτό που συνέβη. *Cause int.*

‘A storm was the cause of what happened.’

β. Δεν υπήρχε συγκεκριμένη αιτία για αυτό που συνέβη. *No cause int.*

‘There was no specific cause for what happened.’

e. *skizo* ‘tear’

17 Active voice – Overt cause context

[Ο Μπάμπης φόρεσε τα καλά του για το πάρτι. Δυστυχώς, καθώς έβγαινε από το σπίτι, η τσέπη του πιάστηκε στο πόμολο.]

‘Babis dressed up for the party. Unfortunately, on leaving the house, his pocket got caught on the doorknob.’

Το παντελόνι έσκισε.

‘The pants teared.’

α. Ένα ατύχημα με το πόμολο ήταν η αιτία για αυτό που συνέβη. *Cause int.*

‘A doorknob accident was the cause of what happened.’

β. Δεν υπήρχε συγκεκριμένη αιτία για αυτό που συνέβη. *No cause int.*

‘There was no specific cause for what happened.’

18 Active voice – Non-overt cause context

[Ο Νίκος ήθελε να βάλει πάλι το γαλάζιο του κοστούμι το παλιό. Μετά από τόση χρήση δεν φοριέται πια.]

‘Nikos wanted to put on his old blue suit again. Having been used for so long, it is not wearable anymore.’

Το παντελόνι έσκισε.

‘The pants teared.’

α. Ένα ατύχημα με το πόμολο ήταν η αιτία για αυτό που συνέβη. *Cause int.*

‘A doorknob accident was the cause of what happened.’

β. Δεν υπήρχε συγκεκριμένη αιτία για αυτό που συνέβη. *No cause int.*

‘There was no specific cause for what happened.’

19 Non-active voice – Overt cause context

[Ο Μπάμπης φόρεσε τα καλά του για το πάρτι. Δυστυχώς, καθώς έβγαινε από το σπίτι, η τσέπη του πιάστηκε στο πόμολο.]

‘Babis dressed up for the party. Unfortunately, on leaving the house, his pocket got caught on the doorknob.’

Το παντελόνι σκίστηκε.

‘The pants teared.’

α. Ένα ατύχημα με το πόμολο ήταν η αιτία για αυτό που συνέβη. *Cause int.*

‘A doorknob accident was the cause of what happened.’

β. Δεν υπήρχε συγκεκριμένη αιτία για αυτό που συνέβη. *No cause int.*

‘There was no specific cause for what happened.’

20 Non-active voice – Non-overt cause context

[Ο Νίκος ήθελε να βάλει πάλι το γαλάζιο του κοστούμι το παλιό. Μετά από τόση χρήση δεν φοριέται πια.]

‘Nikos wanted to put on his old blue suit again. Having been used for so long, it is not wearable anymore.’

Το παντελόνι σκίστηκε.

‘The pants teared.’

α. Ένα ατύχημα με το πόμολο ήταν η αιτία για αυτό που συνέβη. *Cause int.*

‘A doorknob accident was the cause of what happened.’

β. Δεν υπήρχε συγκεκριμένη αιτία για αυτό που συνέβη. *No cause int.*

‘There was no specific cause for what happened.’

f. *madhao* ‘pluck’

21 Active voice – Overt cause context

[Σήμερα θα πάω τα σκυλιά στον κτηνίατρο, χάνουν πολύ τρίχωμα. Πρέπει να έχουν κάτι δερματικό.]

‘Today I am taking the dogs to the vet, they lose too much fur. It must be some skin disease.’

Ο άσπρος σκύλος μάδησε.

‘The white dog plucked.’

α. Μια δερματική ασθένεια ήταν η αιτία για αυτό που συνέβη. *Cause int.*

'A skin disease was the cause of what happened.'

β. Δεν υπήρχε συγκεκριμένη αιτία για αυτό που συνέβη. *No cause int.*

'There was no specific cause for what happened.'

22 Active voice – Non-overt cause context

[Πρέπει να βάλω πάλι ηλεκτρική στο σαλόνι. Ήρθε το μεσημέρι επίσκεψη η Ιωάννα με τα σκυλιά.]

'I need to vacuum the living room again. Ioana visited with the dogs this afternoon.'

Ο άσπρος σκύλος μάδησε.

'The white dog plucked.'

α. Μια δερματική ασθένεια ήταν η αιτία για αυτό που συνέβη. *Cause int.*

'A skin disease was the cause of what happened.'

β. Δεν υπήρχε συγκεκριμένη αιτία για αυτό που συνέβη. *No cause int.*

'There was no specific cause for what happened.'

23 Non-active voice – Overt cause context

[Σήμερα θα πάω τα σκυλιά στον κτηνίατρο, χάνουν πολύ τρίχωμα. Πρέπει να έχουν κάτι δερματικό.]

'Today I am taking the dogs to the vet, they lose too much fur. It must be some skin disease.'

Ο άσπρος σκύλος μαδήθηκε.

'The white dog plucked.'

α. Μια δερματική ασθένεια ήταν η αιτία για αυτό που συνέβη. *Cause int.*

'A skin disease was the cause of what happened.'

β. Δεν υπήρχε συγκεκριμένη αιτία για αυτό που συνέβη. *No cause int.*

‘There was no specific cause for what happened.’

24 Non-active voice – Non-overt cause context

[Πρέπει να βάλω πάλι ηλεκτρική στο σαλόνι. Ήρθε το μεσημέρι επίσκεψη η Ιωάννα με τα σκυλιά.]

‘I need to vacuum the living room again. Ioana visited with the dogs this afternoon.’

Ο άσπρος σκύλος μαδήθηκε.

‘The white dog plucked.’

α. Μια δερματική ασθένεια ήταν η αιτία για αυτό που συνέβη. *Cause int.*

‘A skin disease was the cause of what happened.’

β. Δεν υπήρχε συγκεκριμένη αιτία για αυτό που συνέβη. *No cause int.*

‘There was no specific cause for what happened.’

<u>g. *zarono* ‘wrinkle’</u>

25 Active voice – Overt cause context

[Λόγω της ασθένειάς του είχε μήνες να βγει από το σπίτι. Μόλις αντίκρυσε ξανά τον ήλιο, τα μάτια του πόνεσαν.]

‘Due to his condition, he hadn’t left the house for months. When he saw the sun again, his eyes hurt.’

Το μέτωπό του ζάρωσε.

‘His forehead wrinkled.’

α. Ο ήλιος ήταν η αιτία για αυτό που συνέβη. *Cause int.*

‘The sun was the cause of what happened.’

β. Δεν υπήρχε συγκεκριμένη αιτία για αυτό που συνέβη. *No cause int.*

‘There was no specific cause for what happened.’

26 Active voice – Non-overt cause context

[Είδα τις φωτογραφίες του παππού από τη μέρα του γάμου του. Η αλήθεια είναι ότι δεν άλλαξε και πολύ.]

'I saw grandpa's wedding photos. Truth is he hasn't changed much.'

Το μέτωπό του ζάρωσε.

'His forehead wrinkled.'

α. Ο ήλιος ήταν η αιτία για αυτό που συνέβη. *Cause int.*

'The sun was the cause of what happened.'

β. Δεν υπήρχε συγκεκριμένη αιτία για αυτό που συνέβη. *No cause int.*

'There was no specific cause for what happened.'

27 Non-active voice – Overt cause context

[Λόγω της ασθένειάς του είχε μήνες να βγει από το σπίτι. Μόλις αντίκρυσε ξανά τον ήλιο, τα μάτια του πόνεσαν.]

'Due to his condition, he hadn't left the house for months. When he saw the sun again, his eyes hurt.'

Το μέτωπό του ζαρώθηκε.

'His forehead wrinkled.'

α. Ο ήλιος ήταν η αιτία για αυτό που συνέβη. *Cause int.*

'The sun was the cause of what happened.'

β. Δεν υπήρχε συγκεκριμένη αιτία για αυτό που συνέβη. *No cause int.*

'There was no specific cause for what happened.'

28 Non-active voice – Non-overt cause context

[Είδα τις φωτογραφίες του παππού από τη μέρα του γάμου του. Η αλήθεια είναι ότι δεν άλλαξε και πολύ.]

'I saw grandpa's wedding photos. Truth is he hasn't changed much.'

Το μέτωπό του ζαρώθηκε.

‘His forehead wrinkled.’

α. Ο ήλιος ήταν η αιτία για αυτό που συνέβη. *Cause int.*

‘The sun was the cause of what happened.’

β. Δεν υπήρχε συγκεκριμένη αιτία για αυτό που συνέβη. *No cause int.*

‘There was no specific cause for what happened.’

h. *rayizo* ‘crack’

29 Active voice – Overt cause context

[Η σεισμική δόνηση ήταν πολύ ισχυρή. Το επταώροφο κτήριο κλονίστηκε συθέμελα.]

‘The earthquake was rather strong. The seven-storey building shook to its foundations.’

Οι τοίχοι ράγισαν.

‘The walls cracked.’

α. Μια σεισμική δόνηση ήταν η αιτία για αυτό που συνέβη. *Cause int.*

‘An earthquake was the cause of what happened.’

β. Δεν υπήρχε συγκεκριμένη αιτία για αυτό που συνέβη. *No cause int.*

‘There was no specific cause for what happened.’

30 Active voice – Non-overt cause context

[Μετά από εβδομήντα ολόκληρα χρόνια η πολυκατοικία ήταν πια ακατάλληλη. Τα σημάδια ήταν εμφανή.]

‘After seventy years, the building was now uninhabitable. The signs were obvious.’

Οι τοίχοι ράγισαν.

‘The walls cracked.’

α. Μια σεισμική δόνηση ήταν η αιτία για αυτό που συνέβη. *Cause int.*

‘An earthquake was the cause of what happened.’

β. Δεν υπήρχε συγκεκριμένη αιτία για αυτό που συνέβη. *No cause int.*

‘There was no specific cause for what happened.’

31 Non-active voice – Overt cause context

[Η σεισμική δόνηση ήταν πολύ ισχυρή. Το επταώροφο κτήριο κλονίστηκε συθέμελα.]

‘The earthquake was rather strong. The seven-storey building shook to its foundations.’

Οι τοίχοι ραγίστηκαν.

‘The walls cracked.’

α. Μια σεισμική δόνηση ήταν η αιτία για αυτό που συνέβη. *Cause int.*

‘An earthquake was the cause of what happened.’

β. Δεν υπήρχε συγκεκριμένη αιτία για αυτό που συνέβη. *No cause int.*

‘There was no specific cause for what happened.’

32 Non-active voice – Non-overt cause context

[Μετά από εβδομήντα ολόκληρα χρόνια η πολυκατοικία ήταν πια ακατάλληλη. Τα σημάδια ήταν εμφανή.]

‘After seventy years, the building was now uninhabitable. The signs were obvious.’

Οι τοίχοι ραγίστηκαν.

‘The walls cracked.’

α. Μια σεισμική δόνηση ήταν η αιτία για αυτό που συνέβη. *Cause int.*

‘An earthquake was the cause of what happened.’

β. Δεν υπήρχε συγκεκριμένη αιτία για αυτό που συνέβη. *No cause int.*

‘There was no specific cause for what happened.’

i. *zesteno* ‘heat’

33 Active voice – Overt cause context

[Χωρίς καμία προειδοποίηση το ηφαίστειο εξερράγη. Καυτή λάβα χύθηκε στη γύρω περιοχή, φτάνοντας μέχρι την ακτή.]

‘The volcano erupted all of a sudden. Hot lava spilled over the surrounding area, reaching the coast.’

Η θάλασσα ζέστανε.

‘The sea heated.’

α. Η έκρηξη ενός ηφαιστείου ήταν η αιτία για αυτό που συνέβη. *Cause int.*

‘The eruption of a volcano was the cause of what happened.’

β. Δεν υπήρχε συγκεκριμένη αιτία για αυτό που συνέβη. *No cause int.*

‘There was no specific cause for what happened.’

34 Active voice – Non-overt cause context

[Ηρθε επιτέλους το καλοκαίρι. Οι υποχρεώσεις λιγόστεψαν, η διάθεση ανέβηκε και ο αέρας άρχισε να μυρίζει αλμύρα.]

‘Summer is finally here. The days got calmer, the mood got lighter, and the air got salty.’

Η θάλασσα ζέστανε.

‘The sea heated.’

α. Η έκρηξη ενός ηφαιστείου ήταν η αιτία για αυτό που συνέβη. *Cause int.*

‘The eruption of a volcano was the cause of what happened.’

β. Δεν υπήρχε συγκεκριμένη αιτία για αυτό που συνέβη. *No cause int.*

‘There was no specific cause for what happened.’

35 Non-active voice – Overt cause context

[Χωρίς καμία προειδοποίηση το ηφαίστειο εξερράγη. Καυτή λάβα χύθηκε στη γύρω περιοχή, φτάνοντας μέχρι την ακτή.]

‘The volcano erupted all of a sudden. Hot lava spilled over the surrounding area, reaching the coast.’

Η θάλασσα ζεστάθηκε.

‘The sea heated.’

α. Η έκρηξη ενός ηφαιστείου ήταν η αιτία για αυτό που συνέβη. *Cause int.*

‘The eruption of a volcano was the cause of what happened.’

β. Δεν υπήρχε συγκεκριμένη αιτία για αυτό που συνέβη. *No cause int.*

‘There was no specific cause for what happened.’

36 Non-active voice – Non-overt cause context

[Ήρθε επιτέλους το καλοκαίρι. Οι υποχρεώσεις λιγόστεψαν, η διάθεση ανέβηκε και ο αέρας άρχισε να μυρίζει αλμύρα.]

‘Summer is finally here. The days got calmer, the mood got lighter, and the air got salty.’

Η θάλασσα ζεστάθηκε.

‘The sea heated.’

α. Η έκρηξη ενός ηφαιστείου ήταν η αιτία για αυτό που συνέβη. *Cause int.*

‘The eruption of a volcano was the cause of what happened.’

β. Δεν υπήρχε συγκεκριμένη αιτία για αυτό που συνέβη. *No cause int.*

‘There was no specific cause for what happened.’

j. *tsalakono* ‘crumple’

37 Active voice – Overt cause context

[Μόλις έπιασε το μπουρίνι, η Λίνα έτρεξε να μαζέψει τα πράγματα από το μπαλκόνι. Ξέχασε όμως το βιβλίο με τα παραμύθια της μικρής.]

‘Once the rain started, Lina rushed to pick up the stuff from the balcony. However, she forgot her daughter’s book.’

Το εξώφυλλο τσαλάκωσε.

‘The cover crumpled.’

α. Ένα μπουρίνι ήταν η αιτία για αυτό που συνέβη. *Cause int.*

‘The rain was the cause of what happened.’

β. Δεν υπήρχε συγκεκριμένη αιτία για αυτό που συνέβη. *No cause int.*

‘There was no specific cause for what happened.’

38 Active voice – Non-overt cause context

[Προσπάθησα να διατηρήσω το βιβλίο στην κατάσταση που μου το χάρισε, σαν καινούργιο. Ήταν αδύνατο.]

‘I tried to maintain the book in the condition he gave it to me, as good as new. It was impossible.’

Το εξώφυλλο τσαλάκωσε.

‘The cover crumpled.’

α. Ένα μπουρίνι ήταν η αιτία για αυτό που συνέβη. *Cause int.*

‘The rain was the cause of what happened.’

β. Δεν υπήρχε συγκεκριμένη αιτία για αυτό που συνέβη. *No cause int.*

‘There was no specific cause for what happened.’

39 Non-active voice – Overt cause context

[Μόλις έπιασε το μπουρίνι, η Λίνα έτρεξε να μαζέψει τα πράγματα από το μπαλκόνι. Ξέχασε όμως το βιβλίο με τα παραμύθια της μικρής.]

‘Once the rain started, Lina rushed to pick up the stuff from the balcony. However, she forgot her daughter’s book.’

Το εξώφυλλο τσαλακώθηκε.

‘The cover crumpled.’

α. Ένα μπουρίνι ήταν η αιτία για αυτό που συνέβη. *Cause int.*

‘The rain was the cause of what happened.’

β. Δεν υπήρχε συγκεκριμένη αιτία για αυτό που συνέβη. *No cause int.*

‘There was no specific cause for what happened.’

40 Non-active voice – Non-overt cause context

[Προσπάθησα να διατηρήσω το βιβλίο στην κατάσταση που μου το χάρισε, σαν καινούργιο. Ήταν αδύνατο.]

'I tried to maintain the book in the condition he gave it to me, as good as new. It was impossible.'

Το εξώφυλλο τσαλακώθηκε.

'The cover crumpled.'

α. Ένα μπουρίνι ήταν η αιτία για αυτό που συνέβη. *Cause int.*

'The rain was the cause of what happened.'

β. Δεν υπήρχε συγκεκριμένη αιτία για αυτό που συνέβη. *No cause int.*

'There was no specific cause for what happened.'

Control items

a. change

41 Active voice – Overt cause context

[Η οικονομική κρίση ανέτρεψε τα δεδομένα. Οι κοινωνικός ιστός πρώτα αποδυναμώθηκε και μετά κατέρρευσε.]

'The economic crisis turned everything upside down. The social web got gradually weaker and finally fell apart.'

Το πολιτικό σκηνικό άλλαξε.

'The political stage changed.'

α. Μια οικονομική κρίση ήταν η αιτία για αυτό που συνέβη. *Cause int.*

'An economic crisis was the cause of what happened.'

β. Δεν υπήρχε συγκεκριμένη αιτία για αυτό που συνέβη. *No cause int.*

'There was no specific cause for what happened.'

42 Active voice – Non-overt cause context

[Η αξία της ανθρώπινης ζωής θεωρήθηκε, επιτέλους, αυταπόδεικτη. Στις ψυχές των ανθρώπων άρχισε να καλλιεργείται η δίψα για δημοκρατία.]

‘The value of human life was finally considered self-evident. People started craving for democracy.’

Το πολιτικό σκηνικό άλλαξε.

‘The political stage changed.’

α. Μια οικονομική κρίση ήταν η αιτία για αυτό που συνέβη. *Cause int.*

‘An economic crisis was the cause of what happened.’

β. Δεν υπήρχε συγκεκριμένη αιτία για αυτό που συνέβη. *No cause int.*

‘There was no specific cause for what happened.’

43 Non-active voice – Overt cause context

[Η οικονομική κρίση ανέτρεψε τα δεδομένα. Οι κοινωνικός ιστός πρώτα αποδυναμώθηκε και μετά κατέρρευσε.]

‘The economic crisis turned everything upside down. The social web got gradually weaker and finally fell apart.’

Το πολιτικό σκηνικό μεταβλήθηκε.

‘The political stage changed.’

α. Μια οικονομική κρίση ήταν η αιτία για αυτό που συνέβη. *Cause int.*

‘An economic crisis was the cause of what happened.’

β. Δεν υπήρχε συγκεκριμένη αιτία για αυτό που συνέβη. *No cause int.*

‘There was no specific cause for what happened.’

44 Non-active voice – Non-overt cause context

[Η αξία της ανθρώπινης ζωής θεωρήθηκε, επιτέλους, αυταπόδεικτη. Στις ψυχές των ανθρώπων άρχισε να καλλιεργείται η δίψα για δημοκρατία.]

‘The value of human life was finally considered self-evident. People started craving for democracy.’

Το πολιτικό σκηνικό μεταβλήθηκε.

‘The political stage changed.’

α. Μια οικονομική κρίση ήταν η αιτία για αυτό που συνέβη. *Cause int.*

‘An economic crisis was the cause of what happened.’

β. Δεν υπήρχε συγκεκριμένη αιτία για αυτό που συνέβη. *No cause int.*

‘There was no specific cause for what happened.’

<u>b. sink</u>

45 Active voice – Overt cause context

[Όλοι ήξεραν ότι ήταν πολύ γερό σκαρί. Δυστυχώς, όμως, η καταιγίδα στην οποία έπεσε εκείνο το βράδυ ήταν πολύ ισχυρή.]

‘Everybody knew that it was a well-made ship. Unfortunately, though, the storm it faced that night was too heavy.’

Το καράβι βούλιαξε.

‘The ship sank.’

α. Μια καταιγίδα ήταν η αιτία για αυτό που συνέβη. *Cause int.*

‘A storm was the cause of what happened.’

β. Δεν υπήρχε συγκεκριμένη αιτία για αυτό που συνέβη. *No cause int.*

‘There was no specific cause for what happened.’

46 Active voice – Non-overt cause context

[Οι πειρατές έκλεψαν το φορτίο και απήγαγαν το πλήρωμα. Άφησαν την «Αργώ» άδεια και ακυβέρνητη.]

‘The pirates stole the cargo and abducted the crew. They left “Argo” empty and unmanned.’

Το καράβι βούλιαξε.

‘The ship sank.’

α. Μια καταιγίδα ήταν η αιτία για αυτό που συνέβη. *Cause int.*

'A storm was the cause of what happened.'

β. Δεν υπήρχε συγκεκριμένη αιτία για αυτό που συνέβη. *No cause int.*

'There was no specific cause for what happened.'

47 Non-active voice – Overt cause context

[Όλοι ήξεραν ότι ήταν πολύ γερό σκαρί. Δυστυχώς, όμως, η καταιγίδα στην οποία έπεσε εκείνο το βράδυ ήταν πολύ ισχυρή.]

'Everybody knew that it was a well-made ship. Unfortunately, though, the storm it faced that night was too heavy.'

Το καράβι βυθίστηκε.

'The ship sank.'

α. Μια καταιγίδα ήταν η αιτία για αυτό που συνέβη. *Cause int.*

'A storm was the cause of what happened.'

β. Δεν υπήρχε συγκεκριμένη αιτία για αυτό που συνέβη. *No cause int.*

'There was no specific cause for what happened.'

48 Non-active voice – Non-overt cause context

[Οι πειρατές έκλεψαν το φορτίο και απήγαγαν το πλήρωμα. Άφησαν την «Αργώ» άδεια και ακυβέρνητη.]

'The pirates stole the cargo and abducted the crew. They left "Argo" empty and unmanned.'

Το καράβι βυθίστηκε.

'The ship sank.'

α. Μια καταιγίδα ήταν η αιτία για αυτό που συνέβη. *Cause int.*

'A storm was the cause of what happened.'

β. Δεν υπήρχε συγκεκριμένη αιτία για αυτό που συνέβη. *No cause int.*

‘There was no specific cause for what happened.’

c. turn over

49 Active voice – Overt cause context

[Η καταδίωξη δεν πήγε όπως περιμέναμε. Ο φυγάς έχασε τον έλεγχο και έπεσε με μεγάλη ταχύτητα πάνω σε έναν μεταλλικό φράχτη.]

‘The chase didn’t go as planned. The fugitive lost control and fell at full speed on a metal fence.’

Το όχημα αναποδογύρισε.

‘The vehicle turned over.’

α. Η πρόσκρουση σε έναν φράχτη ήταν η αιτία για αυτό που συνέβη. *Cause int.*

‘Falling on a fence was the cause of what happened.’

β. Δεν υπήρχε συγκεκριμένη αιτία για αυτό που συνέβη. *No cause int.*

‘There was no specific cause for what happened.’

50 Active voice – Non-overt cause context

[Η επίδειξη του νέου αμορτισέρ ήταν μια αποτυχία. Η κατάβαση από την πλαγιά ήταν ανώμαλη και επικίνδυνη.]

‘The demonstration of the new amortisseur was a failure. The descent off the slope was rough and dangerous.’

Το όχημα αναποδογύρισε.

‘The vehicle turned over.’

α. Η πρόσκρουση σε έναν φράχτη ήταν η αιτία για αυτό που συνέβη. *Cause int.*

‘Falling on a fence was the cause of what happened.’

β. Δεν υπήρχε συγκεκριμένη αιτία για αυτό που συνέβη. *No cause int.*

‘There was no specific cause for what happened.’

51 Non-active voice – Overt cause context

[Η καταδίωξη δεν πήγε όπως περιμέναμε. Ο φυγάς έχασε τον έλεγχο και έπεσε με μεγάλη ταχύτητα πάνω σε έναν μεταλλικό φράχτη.]

'The chase didn't go as planned. The fugitive lost control and fell at full speed on a metal fence.'

Το όχημα ανατράπηκε.

'The vehicle turned over.'

α. Η πρόσκρουση σε έναν φράχτη ήταν η αιτία για αυτό που συνέβη. *Cause int.*

'Falling on a fence was the cause of what happened.'

β. Δεν υπήρχε συγκεκριμένη αιτία για αυτό που συνέβη. *No cause int.*

'There was no specific cause for what happened.'

52 Non-active voice – Non-overt cause context

[Η επίδειξη του νέου αμορτισέρ ήταν μια αποτυχία. Η κατάβαση από την πλαγιά ήταν ανώμαλη και επικίνδυνη.]

'The demonstration of the new amortisseur was a failure. The descent off the slope was rough and dangerous.'

Το όχημα ανατράπηκε.

'The vehicle turned over.'

α. Η πρόσκρουση σε έναν φράχτη ήταν η αιτία για αυτό που συνέβη. *Cause int.*

'Falling on a fence was the cause of what happened.'

β. Δεν υπήρχε συγκεκριμένη αιτία για αυτό που συνέβη. *No cause int.*

'There was no specific cause for what happened.'

<u>d. rotate</u>

53 Active voice – Overt cause context

[Ένας κομήτης ασύλληπτων διαστάσεων βρέθηκε στα όρια του ηλιακού μας συστήματος. Το βαρυτικό πεδίο διαταράχθηκε.]

‘A huge comet passed by the borders of our solar system. The gravitational field was disrupted.’

Η Γη γύρισε μία φορά γύρω από τον Ήλιο.

‘The Earth rotated once around the Sun.’

α. Ένας κομήτης ήταν η αιτία για αυτό που συνέβη. *Cause int.*

‘A comet was the cause of what happened.’

β. Δεν υπήρχε συγκεκριμένη αιτία για αυτό που συνέβη. *No cause int.*

‘There was no specific cause for what happened.’

54 Active voice – Non-overt cause context

[Οι εξελίξεις έτρεχαν στη Διαγαλαξιακή Ομοσπονδία. Οι Γήινοι έπρεπε να ψηφίσουν πριν τη λήξη της προθεσμίας.]

‘A lot of things were happening at the Intergalactic Federation. The Earhthlings had to vote before the end of the deadline.’

Η Γη γύρισε μία φορά γύρω από τον Ήλιο.

‘The Earth rotated once around the Sun.’

α. Ένας κομήτης ήταν η αιτία για αυτό που συνέβη. *Cause int.*

‘A comet was the cause of what happened.’

β. Δεν υπήρχε συγκεκριμένη αιτία για αυτό που συνέβη. *No cause int.*

‘There was no specific cause for what happened.’

55 Non-active voice – Overt cause context

[Ένας κομήτης ασύλληπτων διαστάσεων βρέθηκε στα όρια του ηλιακού μας συστήματος. Το βαρυτικό πεδίο διαταράχθηκε.]

‘A huge comet passed by the borders of our solar system. The gravitational field was disrupted.’

Η Γη περιστράφηκε μία φορά γύρω από τον Ήλιο.

‘The Earth rotated once around the Sun.’

α. Ένας κομήτης ήταν η αιτία για αυτό που συνέβη. *Cause int.*

‘A comet was the cause of what happened.’

β. Δεν υπήρχε συγκεκριμένη αιτία για αυτό που συνέβη. *No cause int.*

‘There was no specific cause for what happened.’

56 Non-active voice – Non-overt cause context

[Οι εξελίξεις έτρεχαν στη Διαγαλαξιακή Ομοσπονδία. Οι Γήινοι έπρεπε να ψηφίσουν πριν τη λήξη της προθεσμίας.]

‘A lot of things were happening at the Intergalactic Federation. The Earhthlings had to vote before the end of the deadline.’

Η Γη περιστράφηκε μία φορά γύρω από τον Ήλιο.

‘The Earth rotated once around the Sun.’

α. Ένας κομήτης ήταν η αιτία για αυτό που συνέβη. *Cause int.*

‘A comet was the cause of what happened.’

β. Δεν υπήρχε συγκεκριμένη αιτία για αυτό που συνέβη. *No cause int.*

‘There was no specific cause for what happened.’

<u>e. diminish</u>

57 Active voice – Overt cause context

[Τα πράγματα έμοιαζαν να πηγαίνουν καλύτερα. Ως αποτέλεσμα της αγωγής, όμως, κάποια όργανα άρχισαν να υπολειτουργούν.]

‘Things were seemingly getting better. However, as a result of the medical treatment, some organs started failing.’

Ο χρόνος που του απέμενε λιγόστεψε.

‘The time he had left diminished.’

α. Μια φαρμακευτική αγωγή ήταν η αιτία για αυτό που συνέβη. *Cause int.*

‘A medical treatment was the cause of what happened.’

β. Δεν υπήρχε συγκεκριμένη αιτία για αυτό που συνέβη. *No cause int.*

‘There was no specific cause for what happened.’

58 Active voice – Non-overt cause context

[Είχε μάθει να ζει μία ζωή μέσα στη δράση, χωρίς να νοιάζεται για κανέναν και τίποτα. Όμως τα χρόνια πέρασαν.]

‘He had lived a life full of action, without caring about anybody or anything. But the years passed.’

Ο χρόνος που του απέμενε λιγόστεψε.

‘The time he had left diminished.’

α. Μια φαρμακευτική αγωγή ήταν η αιτία για αυτό που συνέβη. *Cause int.*

‘A medical treatment was the cause of what happened.’

β. Δεν υπήρχε συγκεκριμένη αιτία για αυτό που συνέβη. *No cause int.*

‘There was no specific cause for what happened.’

59 Non-active voice – Overt cause context

[Τα πράγματα έμοιαζαν να πηγαίνουν καλύτερα. Ως αποτέλεσμα της αγωγής, όμως, κάποια όργανα άρχισαν να υπολειτουργούν.]

‘Things were seemingly getting better. However, as a result of the medical treatment, some organs started failing.’

Ο χρόνος που του απέμενε μειώθηκε.

‘The time he had left diminished.’

α. Μια φαρμακευτική αγωγή ήταν η αιτία για αυτό που συνέβη. *Cause int.*

‘A medical treatment was the cause of what happened.’

β. Δεν υπήρχε συγκεκριμένη αιτία για αυτό που συνέβη. *No cause int.*

‘There was no specific cause for what happened.’

60 Non-active voice – Non-overt cause context

[Είχε μάθει να ζει μία ζωή μέσα στη δράση, χωρίς να νοιάζεται για κανέναν και τίποτα. Όμως τα χρόνια πέρασαν.]

‘He had lived a life full of action, without caring about anybody or anything. But the years passed.’

Ο χρόνος που του απέμενε μειώθηκε.

‘The time he had left diminished.’

α. Μια φαρμακευτική αγωγή ήταν η αιτία για αυτό που συνέβη. *Cause int.*

‘A medical treatment was the cause of what happened.’

β. Δεν υπήρχε συγκεκριμένη αιτία για αυτό που συνέβη. *No cause int.*

‘There was no specific cause for what happened.’

<u>f. deteriorate</u>

61 Active voice – Overt cause context

[Πολέμησε με πολύ θάρρος την αρρώστια. Ωστόσο η έκθεση στην πειραματική θεραπεία εξάντλησε τον οργανισμό της.]

‘She fought her disease valiantly. However, the exposure to the experimental treatment exhausted her.’

Η υγεία της χειροτέρεψε.

‘Her health deteriorated.’

α. Μια πειραματική θεραπεία ήταν η αιτία για αυτό που συνέβη. *Cause int.*

‘An experimental treatment was the cause of what happened.’

β. Δεν υπήρχε συγκεκριμένη αιτία για αυτό που συνέβη. *No cause int.*

‘There was no specific cause for what happened.’

62 Active voice – Non-overt cause context

[Υποβλήθηκε σε μία απλή επέμβαση. Ωστόσο, ο οργανισμός της δεν επέτρεψε στη συνοδευτική αγωγή να δράσει.]

‘She had a routine surgery. However, her body did not allow the complementary treatment to work.’

Η υγεία της χειροτέρεψε.

‘Her health deteriorated.’

α. Μια πειραματική θεραπεία ήταν η αιτία για αυτό που συνέβη. *Cause int.*

‘An experimental treatment was the cause of what happened.’

β. Δεν υπήρχε συγκεκριμένη αιτία για αυτό που συνέβη. *No cause int.*

‘There was no specific cause for what happened.’

63 Non-active voice – Overt cause context

[Πολέμησε με πολύ θάρρος την αρρώστια. Ωστόσο η έκθεση στην πειραματική θεραπεία εξάντλησε τον οργανισμό της.]

‘She fought her disease valiantly. However, the exposure to the experimental treatment exhausted her.’

Η υγεία της επιδεινώθηκε.

‘Her health deteriorated.’

α. Μια πειραματική θεραπεία ήταν η αιτία για αυτό που συνέβη. *Cause int.*

‘An experimental treatment was the cause of what happened.’

β. Δεν υπήρχε συγκεκριμένη αιτία για αυτό που συνέβη. *No cause int.*

‘There was no specific cause for what happened.’

64 Non-active voice – Non-overt cause context

[Υποβλήθηκε σε μία απλή επέμβαση. Ωστόσο, ο οργανισμός της δεν επέτρεψε στη συνοδευτική αγωγή να δράσει.]

‘She had a routine surgery. However, her body did not allow the complementary treatment to work.’

Η υγεία της επιδεινώθηκε.

‘Her health deteriorated.’

α. Μια πειραματική θεραπεία ήταν η αιτία για αυτό που συνέβη. *Cause int.*

‘An experimental treatment was the cause of what happened.’

β. Δεν υπήρχε συγκεκριμένη αιτία για αυτό που συνέβη. *No cause int.*

‘There was no specific cause for what happened.’

g. improve

65 Active voice – Overt cause context

[Ο παππούς γκρίνιαζε για μέρες ότι δεν βλέπει από το ένα μάτι. Του πήγα ένα βασικό κολλύριο από το φαρμακείο.]

‘Grandpa was complaining for days that he couldn’t see from one eye. I got him a basic collyrium from the pharmacy.’

Η όρασή του καλυτέρεψε.

‘His vision improved.’

α. Ένα κολλύριο ήταν η αιτία για αυτό που συνέβη. *Cause int.*

‘A collyrium was the cause of what happened.’

β. Δεν υπήρχε συγκεκριμένη αιτία για αυτό που συνέβη. *No cause int.*

‘There was no specific cause for what happened.’

66 Active voice – Non-overt cause context

[Το κουτάβι ήταν τόσο μικρό που δεν μπορούσε καλά καλά να δει. Οι εβδομάδες πέρασαν.]

‘The puppy was so young he couldn’t even see. Weeks passed.’

Η όρασή του καλυτέρεψε.

‘His vision improved.’

α. Ένα κολλύριο ήταν η αιτία για αυτό που συνέβη. *Cause int.*

‘A collyrium was the cause of what happened.’

β. Δεν υπήρχε συγκεκριμένη αιτία για αυτό που συνέβη. *No cause int.*

‘There was no specific cause for what happened.’

67 Non-active voice – Overt cause context

[Ο παππούς γκρίνιαζε για μέρες ότι δεν βλέπει από το ένα μάτι. Του πήγα ένα βασικό κολλύριο από το φαρμακείο.]

‘Grandpa was complaining for days that he couldn’t see from one eye. I got him a basic collyrium from the pharmacy.’

Η όρασή του βελτιώθηκε.

‘His vision improved.’

α. Ένα κολλύριο ήταν η αιτία για αυτό που συνέβη. *Cause int.*

‘A collyrium was the cause of what happened.’

β. Δεν υπήρχε συγκεκριμένη αιτία για αυτό που συνέβη. *No cause int.*

‘There was no specific cause for what happened.’

68 Non-active voice – Non-overt cause context

[Το κουτάβι ήταν τόσο μικρό που δεν μπορούσε καλά καλά να δει. Οι εβδομάδες πέρασαν.]

‘The puppy was so young he couldn’t even see. Weeks passed.’

Η όρασή του βελτιώθηκε.

‘His vision improved.’

α. Ένα κολλύριο ήταν η αιτία για αυτό που συνέβη. *Cause int.*

‘A collyrium was the cause of what happened.’

β. Δεν υπήρχε συγκεκριμένη αιτία για αυτό που συνέβη. *No cause int.*

‘There was no specific cause for what happened.’

h. grow

69 Active voice – Overt cause context

[Ένα χρυσό λουλούδι φύτρωσε ανάμεσα στα αγριόχορτα και τους θάμνους. Οι βροχές του φθινοπώρου το έθρεψαν.]

'A golden flower sprang up among the grass and the bushes. The autumn rain nourished it.'

Ο τρυφερός βλαστός μεγάλωσε.

'The young stem grew.'

α. Οι βροχές ήταν η αιτία για αυτό που συνέβη. *Cause int.*

'The rain was the cause of what happened.'

β. Δεν υπήρχε συγκεκριμένη αιτία για αυτό που συνέβη. *No cause int.*

'There was no specific cause for what happened.'

70 Active voice – Non-overt cause context

[Ο σπόρος της αμυγδαλιάς ταξίδεψε με τον αέρα στο διπλανό χωράφι. Φύτρωσε ανάμεσα στις γέρικες κερασιές.]

'The almond seed travelled through the air to the nearby field. It sprang up between the old cherry trees.'

Ο τρυφερός βλαστός μεγάλωσε.

'The young stem grew.'

α. Οι βροχές ήταν η αιτία για αυτό που συνέβη. *Cause int.*

'The rain was the cause of what happened.'

β. Δεν υπήρχε συγκεκριμένη αιτία για αυτό που συνέβη. *No cause int.*

'There was no specific cause for what happened.'

71 Non-active voice – Overt cause context

[Ένα χρυσό λουλούδι φύτρωσε ανάμεσα στα αγριόχορτα και τους θάμνους. Οι βροχές του φθινοπώρου το έθρεψαν.]

‘A golden flower sprang up among the grass and the bushes. The autumn rain nourished it.’

Ο τρυφερός βλαστός αναπτύχθηκε.

‘The young stem grew.’

α. Οι βροχές ήταν η αιτία για αυτό που συνέβη. *Cause int.*

‘The rain was the cause of what happened.’

β. Δεν υπήρχε συγκεκριμένη αιτία για αυτό που συνέβη. *No cause int.*

‘There was no specific cause for what happened.’

72 Non-active voice – Non-overt cause context

[Ο σπόρος της αμυγδαλιάς ταξίδεψε με τον αέρα στο διπλανό χωράφι. Φύτρωσε ανάμεσα στις γέρικες κερασιές.]

‘The almond seed travelled through the air to the nearby field. It sprang up between the old cherry trees.’

Ο τρυφερός βλαστός αναπτύχθηκε.

‘The young stem grew.’

α. Οι βροχές ήταν η αιτία για αυτό που συνέβη. *Cause int.*

‘The rain was the cause of what happened.’

β. Δεν υπήρχε συγκεκριμένη αιτία για αυτό που συνέβη. *No cause int.*

‘There was no specific cause for what happened.’

<u>i. go crazy</u>

73 Active voice – Overt cause context

[Δεν ξεπέρασε ποτέ το χαμό του γιου του. Έβγαινε τα βράδια και περπατούσε όλο το χωριό ξυπόλυτος φωνάζοντας.]

‘He never got over his son’s death. He would go out at night, walk all over the village barefoot and shout.’

Οι χωριανοί έλεγαν ότι σάλεψε.

‘The villagers thought he went crazy.’

α. Ο θάνατος ενός συγγενή του ήταν η αιτία για αυτό που συνέβη. *Cause int.*

‘The death of a relative was the cause of what happened.’

β. Δεν υπήρχε συγκεκριμένη αιτία για αυτό που συνέβη. *No cause int.*

‘There was no specific cause for what happened.’

74 Active voice – Non-overt cause context

[Υπήρχε ιστορικό ψυχικής ασθένειας στην οικογένεια. Στην προεφηβεία άρχισε και ο μικρός να έχει κάποια επεισόδια.]

‘There was history of mental illness in the family. In pre-adolescence, the boy started having some episodes, too.’

Οι χωριανοί έλεγαν ότι σάλεψε.

‘The villagers thought he went crazy.’

α. Ο θάνατος ενός συγγενή του ήταν η αιτία για αυτό που συνέβη. *Cause int.*

‘The death of a relative was the cause of what happened.’

β. Δεν υπήρχε συγκεκριμένη αιτία για αυτό που συνέβη. *No cause int.*

‘There was no specific cause for what happened.’

75 Non-active voice – Overt cause context

[Δεν ξεπέρασε ποτέ το χαμό του γιου του. Έβγαινε τα βράδια και περπατούσε όλο το χωριό ξυπόλυτος φωνάζοντας.]

‘He never got over his son’s death. He would go out at night, walk all over the village barefoot and shout.’

Οι χωριανοί έλεγαν ότι τρελάθηκε.

‘The villagers thought he went crazy.’

α. Ο θάνατος ενός συγγενή του ήταν η αιτία για αυτό που συνέβη. *Cause int.*

‘The death of a relative was the cause of what happened.’

β. Δεν υπήρχε συγκεκριμένη αιτία για αυτό που συνέβη. *No cause int.*

‘There was no specific cause for what happened.’

76 Non-active voice – Non-overt cause context

[Υπήρχε ιστορικό ψυχικής ασθένειας στην οικογένεια. Στην προεφηβεία άρχισε και ο μικρός να έχει κάποια επεισόδια.]

‘There was history of mental illness in the family. In pre-adolescence, the boy started having some episodes, too.’

Οι χωριανοί έλεγαν ότι τρελάθηκε.

‘The villagers thought he went crazy.’

α. Ο θάνατος ενός συγγενή του ήταν η αιτία για αυτό που συνέβη. *Cause int.*

‘The death of a relative was the cause of what happened.’

β. Δεν υπήρχε συγκεκριμένη αιτία για αυτό που συνέβη. *No cause int.*

‘There was no specific cause for what happened.’

j. explode

77 Active voice – Overt cause context

[Ο εκρηκτικός μηχανισμός απομακρύνθηκε με επιτυχία. Ωστόσο, υπερθερμάνθηκε από τον ήλιο πριν απενεργοποιηθεί.]

‘The explosive device was removed successfully. However, it got overheated from the sun before they could disarm it.

Η βομβα έσκασε.

‘The bomb exploded.’

α. Η υπερθέρμανση του μηχανισμού ήταν η αιτία για αυτό που συνέβη. *Cause int.*

‘The overheating of the device was the cause of what happened.’

β. Δεν υπήρχε συγκεκριμένη αιτία για αυτό που συνέβη. *No cause int.*

‘There was no specific cause for what happened.’

78 Active voice – Non-overt cause context

[Ο αυτοσχέδιος μηχανισμός τοποθετήθηκε στο όχημα. Μόλις η κλεψύδρα άδειασε, ο καταλύτης ήρθε σε επαφή με την καύσιμη ύλη.]

‘The improvised explosive device was put in the vehicle. Once the countdown was over, the catalyst mixed with the fuel.’

Η βομβα έσκασε.

‘The bomb exploded.’

α. Η υπερθέρμανση του μηχανισμού ήταν η αιτία για αυτό που συνέβη. *Cause int.*

‘The overheating of the device was the cause of what happened.’

β. Δεν υπήρχε συγκεκριμένη αιτία για αυτό που συνέβη. *No cause int.*

‘There was no specific cause for what happened.’

79 Non-active voice – Overt cause context

[Ο εκρηκτικός μηχανισμός απομακρύνθηκε με επιτυχία. Ωστόσο, υπερθερμάνθηκε από τον ήλιο πριν απενεργοποιηθεί.]

‘The explosive device was removed successfully. However, it got overheated from the sun before they could disarm it.

Η βόμβα εξερράγη.

‘The bomb exploded.’

α. Η υπερθέρμανση του μηχανισμού ήταν η αιτία για αυτό που συνέβη. *Cause int.*

‘The overheating of the device was the cause of what happened.’

β. Δεν υπήρχε συγκεκριμένη αιτία για αυτό που συνέβη. *No cause int.*

‘There was no specific cause for what happened.’

80 Non-active voice – Non-overt cause context

[Ο αυτοσχέδιος μηχανισμός τοποθετήθηκε στο όχημα. Μόλις η κλεψύδρα άδειασε, ο καταλύτης ήρθε σε επαφή με την καύσιμη ύλη.]

‘The improvised explosive device was put in the vehicle. Once the countdown was over, the catalyst mixed with the fuel.’

Η βόμβα εξερράγη.

‘The bomb exploded.’

α. Η υπερθέρμανση του μηχανισμού ήταν η αιτία για αυτό που συνέβη. *Cause int.*

‘The overheating of the device was the cause of what happened.’

β. Δεν υπήρχε συγκεκριμένη αιτία για αυτό που συνέβη. *No cause int.*

‘There was no specific cause for what happened.’
